# OsDIRP1, a Putative RING E3 Ligase, Plays an Opposite Role in Drought and Cold Stress Responses as a Negative and Positive Factor, Respectively, in Rice (*Oryza sativa* L.)

**DOI:** 10.3389/fpls.2018.01797

**Published:** 2018-12-05

**Authors:** Li Hua Cui, Hye Jo Min, Mi Young Byun, Hyeong Geun Oh, Woo Taek Kim

**Affiliations:** ^1^Department of Systems Biology, College of Life Science and Biotechnology, Yonsei University, Seoul, South Korea; ^2^Institute of Life Science and Biotechnology, Yonsei University, Seoul, South Korea; ^3^Unit of Polar Genomics, Korea Polar Research Institute, Incheon, South Korea

**Keywords:** abscisic acid, cold stress, drought stress, opposite response, rice (*Oryza sativa*), RING E3 Ub ligase

## Abstract

As higher plants are sessile organisms, they are unable to move to more favorable places; thus, they have developed the ability to survive under potentially detrimental conditions. Ubiquitination is a crucial post-translational protein modification and participates in abiotic stress responses in higher plants. In this study, we identified and characterized OsDIRP1 (*Oryza sativa* Drought-Induced RING Protein 1), a nuclear-localized putative RING E3 ubiquitin (Ub) ligase in rice (*Oryza sativa* L.). *OsDIRP1* expression was induced by drought, high salinity, and abscisic acid (ABA) treatment, but not by low temperature (4°C) stress, suggesting that *OsDIRP1* is differentially regulated by different abiotic stresses. To investigate its possible role in abiotic stress responses, *OsDIRP1*-overexpressing transgenic rice plants (*Ubi:OsDIRP1-sGFP*) were generated, and their phenotypes were analyzed. The T4 *Ubi:OsDIRP1-sGFP* lines showed decreased tolerance to drought and salt stress as compared to wild-type rice plants. Moreover, *Ubi:OsDIRP1-sGFP* progeny were less sensitive to ABA than the wild-type during both germination and post-germination growth. In contrast, *Ubi:OsDIRP1-sGFP* plants exhibited markedly higher tolerance to prolonged cold (4°C) treatment. These results suggest that OsDIRP1 acts as a negative regulator during drought and salt stress, whereas it functions as a positive factor during the cold stress response in rice.

## Introduction

As sessile organisms, higher plants are constantly exposed to diverse environmental stresses, such as water deficit, high salinity, and extreme temperatures, throughout their life cycle that they cannot avoid. These detrimental conditions can disrupt plant growth, and on a larger scale, reduce global crop production (Ray et al., 2015). In addition to abiotic stress in plants, rapid increases in population also affect global food security (Lesk et al., 2016). Rice is a primary source of food for more than half of the world’s population. Thus, improving the stress tolerance of rice is crucial to maintain high crop productivity and fulfill the growing demand for food.

Plant stress response and acclimation are regulated by a complex network of cellular factors, including stress perception and related signaling pathways, changes in hormonal and metabolic balances, and transcriptional and post-transcriptional regulation of stress-associated genes (Osakabe et al., 2014; Guerra et al., 2015). Ubiquitination is a post-translational modification of diverse cellular proteins. In higher plants, the ubiquitination pathway is critically involved in the abiotic stress response (Santner and Estelle, 2010; Lyzenga and Stone, 2012; Yu et al., 2016). Ubiquitination is a multistep process, in which ubiquitin (Ub) is conjugated to target proteins by successive reactions catalyzed by three types of enzymes, E1 Ub activating enzymes, E2 Ub conjugating enzymes, and E3 Ub ligases. In general, E3 ligases specifically recognize the target protein (Smalle and Viestra, 2004). E3 Ub ligases are divided into single- and multi-subunit types depending on their structure. Single-subunit E3 Ub ligases are further categorized into distinct families based on the presence of specific domains, such as the really interesting new gene (RING), U-box, and homology to E6AP C terminus (HECT) domains. Skp1-cullin-F-box (SCF) and anaphase-promoting complex (APS) belong to the multi-subunit E3 Ub ligases (Lee and Kim, 2011).

Abscisic acid (ABA) is a well-known plant stress hormone that plays a pivotal role in the response to environmental stimuli (Zhu, 2002; Yamaguchi-Shinozaki and Shinozaki, 2006). Cellular levels of ABA are rapidly increased under stress conditions, which induces various physiological responses, such as stomata closure, growth inhibition, and the production of proline and sugars. Different kinds of E3 Ub ligases participate in both ABA production and the ABA-mediated stress response. For example, the *Arabidopsis* U-box E3 Ub ligase SAUL1/PUB44 negatively regulates the ABA biosynthetic enzyme aldehyde oxidase 3 (AAO3), whereas the RING E3 Ub ligase XERICO promotes ABA production by enhancing the expression of 9-*cis*-epoxycarotenoid dioxygenase 3 (NCED3) (Ko et al., 2006; Raab et al., 2009). In addition, E3 Ub ligases regulate ABA perception and the signal transduction cascade by mediating the degradation of ABA receptors, SnRK protein kinases, and transcription factors (Chen et al., 2013; Lyzenga et al., 2013; Bueso et al., 2014; Irigoyen et al., 2014; Zhao et al., 2017).

RING E3 Ub ligases widely exist in eukaryotic organisms. Compared with *Saccharomyces cerevisiae*, which has 47 RING-type E3 encoding genes, rice and *Arabidopsis* contain 378 and 499 genes, respectively (Kraft et al., 2005; Stone et al., 2005; Mazzucotelli et al., 2006; Li et al., 2008; Du et al., 2009). Recent studies have revealed the cellular roles of RING E3 Ub ligases in response to abiotic stress in the dicot model plant *Arabidopsis* (Cho et al., 2017; Stone, 2018). In contrast, our knowledge concerning the roles of RING E3 ligases in the monocot cereal rice in response to abiotic stresses is relatively rudimentary. SALT-AND DROUGHT-INDUCED RING FINGER1 (SDIR1) is a positive regulator of ABA-mediated drought stress response in *Arabidopsis* (Zhang et al., 2007; Zhang et al., 2015). OsSDIR1 is a rice ortholog of SDIR1 and positively involved in the drought stress response in rice plants (Gao et al., 2011). *Oryza sativa* Drought-Induced SINA protein1 (OsDIS1), a rice RING E3 Ub ligase, plays a negative role in drought stress tolerance through posttranslational regulation of *Oryza sativa* NIMA-related kinase 6 (OsNek6), which is a tubulin complex-related serine/threonine protein kinase, and transcriptional regulation of stress-related genes (Ning et al., 2011). Recently, rice *Oryza sativa* Chloroplast Targeting RING E3 ligase 1 (OsCTR1) and *Oryza sativa* Arsenic-Induced RING E3 ligase 1 (OsAIR1) were identified as positive factors in the drought stress response and post-germination growth under arsenate stress conditions, respectively (Lim et al., 2014; Hwang et al., 2016). Furthermore, knock-down of *Oryza sativa* Stress-Related RING Finger Protein 1 (*OsSRFP1*) resulted in increased tolerance to high salt and cold stress in rice (Fang et al., 2015).

In this study, we identified and characterized *Oryza sativa* Drought-Induced RING Protein 1 (OsDIRP1), which is a nuclear-localized putative RING-type E3 Ub ligase in rice. *OsDIRP1* was induced by drought, high salinity, and ABA treatments, but not by cold stress (4°C). *OsDIRP1*-overexpressing T4 transgenic rice plants (*Ubi:OsDIRP1-sGFP*) exhibited reduced tolerance to drought and salt stress as compared to wild-type rice plants. *Ubi:OsDIRP1-sGFPs* plants were less sensitive to ABA than wild-type plants during both the germination and post-germination stages. In contrast, the *Ubi:OsDIRP1-sGFP* progeny displayed markedly increased tolerance to cold stress relative to the wild-type plants. These results indicate that OsDIRP1 acts as a negative regulator of the drought and salt stress responses, while it works as a positive factor of the cold stress response in rice.

## Materials and Methods

### Plant Materials and Growth Conditions

Dry seeds of the rice (*Oryza sativa* L.) japonica variety ‘Dong-jin’ were washed with 70% ethanol and subsequently sterilized with a 0.4% NaClO solution. Sterilized seeds were germinated and grown on half-strength Murashige and Skoog (MS) medium (Duchefa Biochemie, Haarlem, Netherlands) supplemented with 3% sucrose and 0.75% phytoagar (pH 5.6–5.8) for 10–12 days. Germinated seedlings were transplanted to soil and grown at 25–30°C under long-day conditions (16 h light and 8 h dark) in a greenhouse. Whole seedlings of 10-day-old wild-type rice plants were subjected to drought (dried on filter paper for 0, 0.25, 0.5, 1, 2, and 4 h), salt (soaked in a 200 mM NaCl solution for 0, 1, 4, and 6 h), cold (incubated at 4°C for 0, 12, and 24 h), and 100 μM ABA (0, 3, 6, 12, and 24 h) treatments.

### RNA Extraction, RT-PCR, and Real-Time Quantitative RT-PCR (qRT-PCR) Analyses

Total RNA was extracted from various tissues of wild-type rice plants and stress-treated seedlings by using the Easy Spin Plants Total RNA Extraction kit (iNtRON Biotechnology, Korea) according to the manufacturer’s protocol. RNA was quantified using a spectrophotometer (NanoDrop 1000; Thermo Scientific, United States). Total RNA (3 μg) was used to synthesize cDNA by using TOPscript Reverse Transcriptase (Enzynomics, Korea) and oligo (dT) primers. The PCR amplification procedure was as follows: 5 min of denaturation and enzyme activation at 95°C, followed by 28–33 cycles of 30 s at 95°C, 30 s at 55°C, and 20 s at 72°C. PCR products were separated on a 2% agarose gel and visualized under UV light. The *OsUbiquitin* gene was included as a loading control. *OsRab16b* was used as a positive control for the drought, salt, and ABA treatments, whereas *OsDREB1A* was used as a positive control for cold stress. qRT-PCR was carried out using PikoReal real-time PCR (Thermo Scientific, United States) with SYBR Premix Ex Taq II (Takara, Japan) as described previously (Kim et al., 2017). The primer sequences used for RT-PCR and qRT-PCR are listed in Supplementary Table S1.

### *In vitro* Self-Ubiquitination Assay

An *in vitro* self-ubiquitination assay was performed as described in a previous study (Bae et al., 2011). Bacterially expressed GST-OsDIRP1 recombinant fusion protein was incubated at 30°C for 1 h in ubiquitination reaction buffer (10 mM ATP, 0.5 mM DTT, 2.5 mM MgCl_2,_ 50 mM Tris-HCl, pH 7.5, and 0.5 μg Ub) in the presence or absence of E1 (*Arabidopsis* UBA1) and E2 (*Arabidopsis* UBC8). The reaction products were subjected to immunoblot analysis with anti-GST and anti-Ub antibodies as described previously (Bae and Kim, 2014).

### Subcellular Localization

The full-length coding region of *OsDIRP1* was tagged with synthetic green fluorescent protein (*sGFP*) in-frame and inserted into the pBI221 binary vector. Isolation of rice protoplasts and transfection of the vectors into the protoplasts were performed according to the method of Yoo et al. (2007), with modifications as described by Byun et al. (2017). The *35S:sGFP, 35S:OsDIRP1-sGFP*, and *35S:NLS-mRFP* constructs were transfected or co-transfected into the isolated protoplasts and incubated overnight. The fluorescent signals of the expressed proteins were observed by fluorescence microscopy (BX51; Olympus, Japan) as described previously (Seo et al., 2016). NLS-mRFP was used as a nuclear marker protein.

### Generation of *OsDIRP1*-Overexpressing (*Ubi:OsDIRP1-sGFP*) and RNAi-Mediated Knock-Down (*Ubi:RNAi-OsDIRP1*) Transgenic Rice Plants

The *Ubi:OsDIRP1-sGFP* and *Ubi:RNAi-OsDIRP1* chimeric constructs were transformed into *Agrobacterium tumefaciens* strain LBA4404 via electroporation as described by Park et al. (2016). Transgenic rice plants were produced by using pGA2897 binary vector plasmids that contained the maize ubiquitin promoter (*Ubi*). All rice transformation procedures were performed as described previously by Byun et al. (2017). Generated transgenic rice T0 plants were transplanted in the soil under greenhouse condition and further propagated in the field condition. The harvested transgenic seeds of individual plant were germinated in hygromycin containing media to select the homozygous plants in T2 generation. Independent homozygous T4 *OsDIRP1-*overexpressing (*Ubi:OsDIRP1-sGFP* lines #1, #2, and #3) and homozygous T4 *RNAi* knock-down (*Ubi:RNAi-OsDIRP1* lines #1, #2, and #3) transgenic rice plants were used for phenotypic analyses.

### Genomic DNA Extraction and DNA Gel Blot Analysis

Total genomic DNA was extracted from the leaves of 5-week-old wild-type and transgenic rice plants by using the CTAB (2% CTAB, 20 mM EDTA, 1.4 M NaCl, 2% PVP-40, and 100 mM Tris-HCl, pH 8.0) method. Total genomic DNA was digested with *Hin*dIII restriction enzyme (Thermo Scientific, United States) and separated by electrophoresis on a 0.7% agarose gel. Then, the DNA on the gel was transferred to a Hybond-N nylon membrane (GE Healthcare, United Kingdom) by the capillary transfer method, and the blot was hybridized to a ^32^P-labeled hygromycin B phosphotransferase (*Hph*) probe as described by Byun et al. (2018). The autoradiography signals were visualized using a BAS2500 Bio-Imaging Analyzer (Fuji Film, Japan).

### Phenotype Evaluation of *Ubi:OsDIRP1-sGFP* and *Ubi:RNAi-OsDIRP1* Transgenic Rice Plants Grown in a Paddy Field

The agronomic traits of the T4 *Ubi:OsDIRP1-sGFP* and T4 *Ubi:RNAi-OsDIRP1* transgenic rice plants grown under normal field conditions were analyzed as described by Park et al. (2016). The following agronomic traits were scored: the number of panicles, panicle length, number of primary branches per panicle, 1000-grain weight, and total grain weight.

### Stress Treatment of Wild-Type, *Ubi:OsDIRP1-sGFP*, and *Ubi:RNAi-OsDIRP1* Rice Plants

For the drought stress treatment, 5-week-old wild-type, T4 *Ubi:OsDIRP1-sGFP* (independent lines #1, #2, and #3), and T4 *Ubi:RNAi-OsDIRP1* (independent lines #1, #2, and #3) plants were grown in the same pot without watering for 8–9 days until the leaves were wilted. After 8–9 days of dehydration, the plants were re-watered, and their growth profiles were monitored at different time points during stress recovery. Plants that resumed growing, with green and healthy leaves, were regarded as having survived. The survival rates and total leaf chlorophyll content were determined at 15–20 days of recovery. Data were obtained from at least six independent biological experiments. To measure the rate of leaf water loss, detached leaves from 6-week-old wild-type and transgenic rice plants were placed on a filter paper at room temperature and weighed after different time intervals (15, 30, 60, 90, 120, 180, 240, and 300 min). The water loss rate was calculated as the percentage of the initial fresh weight.

For the salt stress treatment, 5-week-old wild-type and transgenic rice plants, which had been grown in the same pot under normal conditions, were transferred to a tray containing a solution of 200 mM NaCl. After 16–18 days of salt treatment, the plants were recovered by normal irrigation. Plants that resumed growing, with green and healthy leaves, were regarded as having survived. The survival rates and total leaf chlorophyll content were determined after 1 month of recovery. At least five independent biological experiments were performed.

For the cold stress treatment, 5-week-old plants grown in the same pot at 28°C under long-day conditions (16 h light and 8 h dark) were transferred to a cold room at 4°C for 6–8 days, after which the plants were recovered at 28°C for 25–30 days, and their growth patterns were monitored as described previously (Byun et al., 2015). Electrolyte leakage was analyzed using 8-day-old seedlings at different time points before and after cold stress treatment (0, 5, and 10 days at 4°C). Seedlings of wild-type and transgenic plants were soaked in a test tube containing 35 mL of distilled water on an orbital shaker (200 rpm) at 28°C overnight. The electrolyte conductivity of each sample was determined before and after autoclaving by using a conductivity meter (Orion Star A212; Thermo Scientific, United States) according to the method of Min et al. (2016).

### Leaf Disk Assay and Measurement of Total Leaf Chlorophyll Content

Leaf disks (0.5 cm in diameter) prepared from 5-week-old wild-type and transgenic plants were floated in various concentrations (0, 200, 400, 600, and 800 mM) of NaCl for 3 days under long-day conditions (16 h light and 8 h dark). Representative photos were taken after 3 days of incubation, and the total leaf chlorophyll content of each sample was measured.

Total leaf chlorophyll (chlorophyll a + chlorophyll b) content was measured according to Lichtenthaler (1987), with modifications as described by Min et al. (2016). The amounts of chlorophyll a + chlorophyll b were measured at 664.2 nm and 648.6 nm, respectively, with an ELISA microplate reader (VERSAmax, Molecular Devices, United States) and normalized to the dry weight of the leaves of each genotype.

### ABA-Dependent Germination and Post-germination Tests

Wild-type and T4 *Ubi: OsDIRP1-sGFP* transgenic (lines #1, #2, and #3) seeds were germinated and grown on half-strength MS medium supplemented with different concentrations (0, 3, and 5 μM) of ABA (Sigma-Aldrich, Germany) at 28°C under long-day conditions (16 h light and 8 h dark). After 7 days of germination, shoot and root lengths were measured. More than 50 seeds were used in each assay, and three independent biological assays were performed.

For the post-germination assays, wild-type and transgenic seeds were germinated on half-strength MS medium for 2 days. Then, the germinated seedlings were transferred to half-strength MS medium containing 0, 3, 5, or 10 μM ABA and incubated for another 6 days. At least 100 seeds were used in each assay, and four independent biological assays were performed.

## Results

### Identification and Characterization of *OsDIRP1* in Rice

**FIGURE 1:**
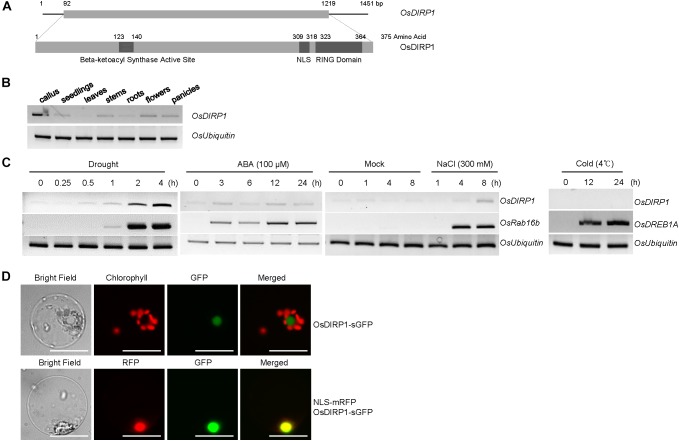
Identification and characterization of *OsDIRP1* in rice. **(A)** (Upper) Schematic diagram of full-length *OsDIRP1* cDNA. The solid bar depicts the coding region. The solid lines represent the 5’- and 3’-untranslated regions. (Lower) Schematic structure of OsDIRP1. The putative beta-ketoacyl synthase active site, nuclear localization sequence (NLS), and RING motif are shown as dark gray bars. **(B)** RT-PCR analysis of *OsDIRP1* in different tissues of rice plants. Total RNA was isolated from various rice tissues as indicated and analyzed by RT-PCR. *OsUbiquitin* was used as an equal loading control. **(C)** Expression patterns of *OsDIRP1* in response to various abiotic stresses in rice plants. Light-grown, 10-day-old wild-type seedlings were subjected to drought, salt, cold, and ABA treatments at different time points as indicated. *OsUbiquitin* was used as an internal control for all the RT-PCR analyses. *OsRab16b* was used as a positive control for drought, salt, and ABA treatments, whereas *OsDREB1A* was used as a positive control for cold stress. **(D)** Subcellular localization of OsDIRP1. A *35S:OsDIRP1-sGFP* fusion construct was transfected into wild-type rice protoplasts, and the fluorescent signals of the expressed proteins were visualized by fluorescence microscopy under dark-field conditions. sGFP and NLS-mRFP were used as cytosol- and nucleus-localized marker proteins, respectively. Bars = 5 μm.

To identify the RING E3 Ub ligases that participate in abiotic stress responses in rice, we analyzed the expression patterns of the RING-type E3 ligase gene family under drought stress based on publicly available rice microarray data^1^. *Os06g47270* was identified to be induced by water deficit and was named *Oryza sativa* Drought-Induced RING Protein 1 (*OsDIRP1*). *OsDIRP1* encodes a 375 amino acid protein with a single C_3_H_2_C_3_-type RING motif in the C-terminal region, a nuclear localization sequence (NLS), and a putative beta-ketoacyl synthase active site in the N-terminal region (Figure 1A). OsDIRP1 was 48%, 47%, and 43% identical to putative RING E3 Ub ligases in monocot millet (*Setaria italica*), maize (*Zea mays*), and sorghum (*Sorghum bicolor*), respectively (Supplementary Figures S1A,B).

*OsDIRP1* transcripts were detected in all examined rice tissues, including the callus, 11-day-old seedlings, developing and mature leaves, stems, roots, flowers, and panicles (Figure 1B). Furthermore, *OsDIRP1* was induced by drought (0.25–4 h), high salt (300 mM NaCl for 1–8 h), and ABA (100 μM for 3–24 h) treatments, but not by cold temperature (4°C for 12 and 24 h) (Figure 1C). The original figures were added in Supplementary Materials indicated as Supplementary Presentation S1.

The subcellular localization of OsDIRP1 was investigated via the protoplast transient expression system. The *35S:OsDIRP1-sGFP* chimeric construct was expressed in wild-type rice protoplasts with or without *35S:NLS-mRFP*, and the expressed proteins were visualized by fluorescence microscopy. The results revealed that the fluorescence signal of OsDIRP1-sGFP was predominantly located in the nucleus, where it overlapped with the nuclear marker protein NLS-mRFP (Figure 1D), which suggests that OsDIRP1 is a nuclear protein.

### Generation and Molecular Characterization of *OsDIRP1*-Overexpressing and *RNAi*-Mediated Knock-Down Transgenic Rice Plants

**FIGURE 2:**
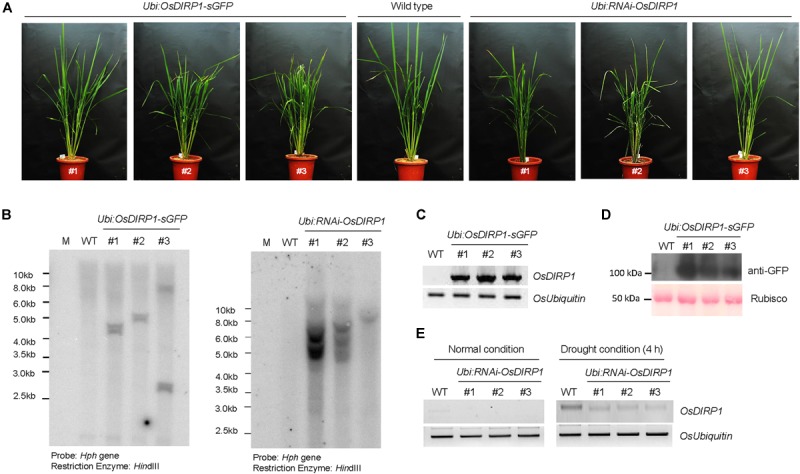
Molecular characterization of *OsDIRP1-*overexpressing and *RNAi*-mediated knock-down transgenic rice plants. **(A)** Morphology of 2-month-old wild-type (WT), T4 *Ubi:OsDIRP1-sGFP*, and T4 *Ubi:RNAi-OsDIRP1* rice plants grown under long-day conditions (16 h light and 8 h dark). **(B)** Genomic Southern blot analysis. Total leaf genomic DNA was isolated from wild-type (WT), T4 *Ubi:OsDIRP1-sGFP* (lines #1, #2, and #3), and T4 *Ubi:RNAi-OsDIRP1* (lines #1, #2, and #3) rice plants. The DNA was digested with *Hin*dIII and hybridized to a ^32^P-labeled hygromycin B phosphotransferase (*Hph*) probe under high stringency conditions. **(C)** RT-PCR analysis of the wild-type (WT) and T4 *Ubi:OsDIRP1-sGFP* (independent lines #1, #2, and #3) transgenic rice plants to examine *OsDIRP1* transcript levels. *OsUbiquitin* was used as a loading control. **(D)** Immunoblot analysis of wild-type (WT) and T4 *Ubi:OsDIRP1-sGFP* plants. Total proteins were isolated using 2x SDS sample buffer and immunoblotted with anti-GFP antibody. Rubisco was used as an equal loading control. **(E)** RT-PCR analysis of the wild-type (WT) and T4 *Ubi:RNAi-OsDIRP1* plants. RNA was isolated from whole seedlings of non-drought-treated (0 h) and drought-treated (4 h) wild-type (WT) and *Ubi:RNAi-OsDIRP1* (lines #1, #2, and #3) plants. *OsUbiquitin* was used as a loading control.

To investigate the cellular role of OsDIRP1 in abiotic stress responses, *OsDIRP1*-overexpressing (*Ubi:OsDIRP1-sGFP*) and *RNAi*-mediated *OsDIRP1* knock-down (*Ubi:RNAi-OsDIRP1*) transgenic rice plants were generated. Under normal growth conditions, there was no detectable morphological difference between the wild-type and transgenic rice plants (Figure 2A). The results of genomic Southern blot analysis showed that there were three independent lines of each genotype (Figure 2B). In the *OsDIRP1*-overexpressing transgenic lines, the amount of *OsDIRP1-sGFP* transcript was markedly increased as compared to the level in wild-type plants under normal growth conditions as measured by RT-PCR (Figure 2C). Overexpression of OsDIRP1-sGFP protein was confirmed by immunoblot analysis with an anti-GFP antibody (Figure 2D). The original figures were added in Supplementary Materials indicated as Supplementary Presentation S1. The level of *OsDIRP1* transcript was partially suppressed in the *Ubi:RNAi-OsDIRP1* knock-down lines relative to that in the wild-type plants under both normal and drought conditions (Figure 2E). The agronomic traits of *Ubi:OsDIRP1-sGFP* and *Ubi:RNAi-OsDIRP1* T4 progeny grown in a paddy field condition were analyzed with respect to the number of panicles, panicle length, number of primary branches per panicle, 1000-grain weight, and total grain weight. As shown in Table 1 and Supplementary Figure S2, there was no significant difference among the wild-type, *Ubi:OsDIRP1-sGFP*, and *Ubi:RNAi-OsDIRP1* plants. Thus, overexpression and knock-down of *OsDIRP1* did not alter growth during the vegetative and reproductive stages. These *OsDIRP1*-overexpressing and *RNAi*-mediated *OsDIRP1* knock-down T4 transgenic plants were used for subsequent phenotypic analysis of their response to abiotic stress.

**Table 1:** Agronomic traits of the wild-type (WT), T4 *Ubi:OsDIRP1-sGFP* (OE, independent lines #1, #2, and #3), and T4 *Ubi:RNAi-OsDIRP1* (KD, independent lines #1, #2, and #3) rice plants grown in the paddy field conditions.

Agronomic traits	WT	OE#1	OE#2	OE#3	KD#1	KD#2	KD#3
Number of panicle per hill	14 ± 2	15 ± 2	16 ± 2	16 ± 4	14 ± 2	16 ± 2	15 ± 3
Panicle length (cm)	20.01 ± 0.81	17.98 ± 1.09	17.18 ± 0.87	17.73 ± 1.25	20.05 ± 0.73	17.78 ± 0.53	18.40 ± 1.28
Number of primary branch per panicle	11 ± 1	11 ± 1	10 ± 1	10 ± 1	12 ± 1	10 ± 1	11 ± 1
1000-grain weight (g)	26.05 ± 0.30	28.18 ± 0.75	26.95 ± 0.85	28.00 ± 1.70	28.82 ± 1.13	27.63 ± 0.72	28.98 ± 0.70
Total grain weight (g)	38.50 ± 6.79	40.31 ± 6.35	40.07 ± 7.85	36.51 ± 7.65	34.55 ± 5.07	38.92 ± 6.82	34.30 ± 8.80


*Data are shown as mean ± SD from 10 plants of each genotype.*

### *OsDIRP1*-Overexpressing Transgenic Rice Plants Exhibited Reduced Tolerance to Drought and Salt Stress Compared to Wild-Type Plants

**FIGURE 3:**
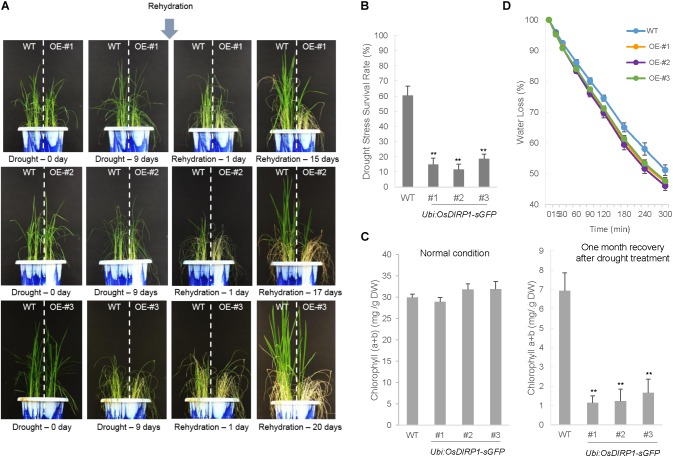
Decreased tolerance of *Ubi:OsDIRP1-sGFP* transgenic rice plants to drought stress. **(A)** Drought stress phenotypes of wild-type and T4 *Ubi:OsDIRP1-sGFP* transgenic rice plants. Light-grown, 5-week-old wild-type (WT) and T4 *Ubi:OsDIRP1-sGFP* (lines #1, #2, and #3) plants were grown for 9 days without watering (drought stress). After 9 days of dehydration, these plants were re-watered, and their growth patterns were monitored for 15–20 days after re-watering. OE represents *OsDIRP1*-overexpressing transgenic rice plants. **(B)** Survival rates of the wild-type (WT) and T4 *Ubi:OsDIRP1-sGFP* plants in response to drought stress. Data are means ± SE (*n* ≥ 6 independent biological experiments; >30 plants were used in each assay, ^∗∗^*P* < 0.01, Student’s *t*-test). **(C)** Total leaf chlorophyll content of wild-type and T4 *Ubi:OsDIRP1-sGFP* (lines #1, #2, and #3) plants before and after drought treatment. The chlorophyll content of mock-treated (before drought) and drought-treated plants was measured after 1 month of rehydration. Data are means ± SE (*n* ≥ 3 independent biological experiments; >30 plants were used in each assay, ^∗∗^*P* < 0.01, Student’s *t*-test). **(D)** Water loss rates of detached leaves. The leaves of 5-week-old wild-type and T4 *Ubi:OsDIRP1-sGFP* (lines #1, #2, and #3) plants were detached, and their fresh weights were measured at the indicated time points. Data are means ± SD (*n* = 3 independent biological experiments; >6 plants of each genotype were used in each experiment).

Wild-type and *Ubi:OsDIRP1-sGFP* T4 transgenic (independent lines #1, #2, and #3) rice plants were grown at 28°C for 5 weeks under long-day conditions (16 h light and 8 h dark). The plants were then subjected to drought stress by withholding water for 9 days. After 9 days of dehydration, these plants were re-watered, and their growth patterns were monitored for 15–20 days after initiation of re-watering. Of the wild-type rice plants, 61.0 ± 5.7% resumed growth after rehydration (Figure 3A). In contrast, most of the *Ubi:OsDIRP1-sGFP* lines exhibited pale green and yellowish leaves, and only 10.7 ± 2.9% – 19.0 ± 3.0% of the *OsDIRP1*-overexpressing lines survived after rehydration (Figure 3B).

Mature leaves were detached from plants of each genotype to measure the chlorophyll content (chlorophyll a + chlorophyll b) before and after the drought treatment. Before drought treatment, the total leaf chlorophyll amounts of the wild-type and *Ubi:OsDIRP1-sGFP* plants were indistinguishable. However, the *OsDIRP1*-overexpressing progeny contained much lower amounts of chlorophyll (1.1 ± 0.4 – 1.6 ± 0.7 mg/g DW) as compared to those of the wild-type plants (6.9 ± 0.9 mg/g DW) after recovery from drought treatment (Figure 3C). In addition, the detached leaves of *OsDIRP1*-overexpressors lost their water content faster than the wild-type leaves. After a 3-h incubation at room temperature, wild-type and *Ubi:OsDIRP1-sGFP* leaves lost 58.1 ± 4.1% and 59.5 ± 3.3% – 61.3 ± 1.8% of their fresh weight, respectively (Figure 3D). After a 6-h incubation, 51.2 ± 3.5% and 46.0 ± 3.0% – 47.7 ± 2.2% of the initial fresh weight were retained in the wild-type and *Ubi:OsDIRP1-sGFP* leaves, respectively (Figure 3D). Thus, overexpression of *OsDIRP1* resulted in reduced tolerance to dehydration stress, indicating that OsDIRP1 negatively influences the drought stress response in rice.

**FIGURE 4:**
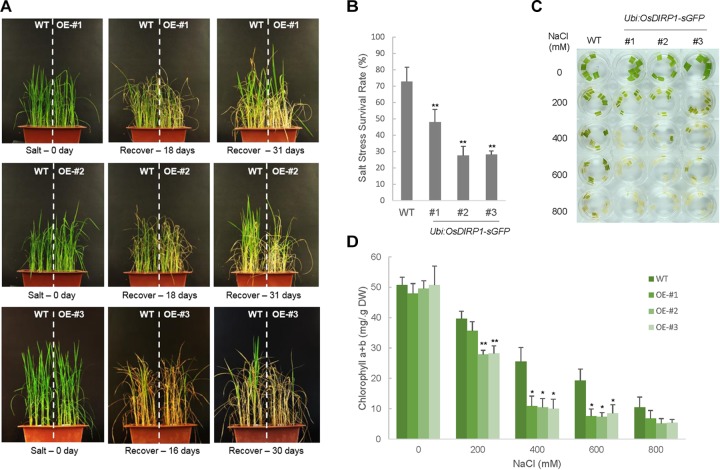
Decreased tolerance of *Ubi:OsDIRP1-sGFP* transgenic rice plants in response to salt stress. **(A)** The salt stress phenotypes of wild-type and T4 *Ubi:OsDIRP1-sGFP* plants. Light-grown, 5-week-old wild-type and T4 *Ubi:OsDIRP1-sGFP* (lines #1, #2, and #3) plants were treated with 200 mM NaCl for 16–18 days and then transferred to normal growth conditions with watering for 1 month. OE represents *OsDIRP1*-overexpressing transgenic rice plants. **(B)** Survival rates of wild-type (WT) and T4 *Ubi:OsDIRP1-sGFP* plants in response to salt stress. Data are means ± SE (*n* ≥ 5 independent biological experiments; >40 plants were used in each assay, ^∗^*P* < 0.05, ^∗∗^*P* < 0.01, Student’s *t*-test). **(C)** Leaf disk senescence assays in response to high salinity. Leaf disks (0.5 cm in diameter) were prepared from 5-week-old wild-type and transgenic plants and floated in different concentrations (0, 200, 400, 600, and 800 mM) of NaCl for 3 days. A representative photo was taken after 3 days of incubation. **(D)** Chlorophyll content in the leaf disk senescence assay. The amounts of chlorophyll (chlorophyll a + chlorophyll b) in the wild-type and T4 *Ubi:OsDIRP1-sGFP* (lines #1, #2, and #3) leaf disks were determined 3 days after incubation with different concentrations (0, 200, 400, 600, and 800 mM) of NaCl. Data are means ± SE (*n* ≥ 4 independent biological experiments, ^∗∗^*P* < 0.01, Student’s *t*-test).

Because *OsDIRP1* was induced by high salinity (Figure 1B), the salt tolerance of the *Ubi:OsDIRP1-sGFP* plants was evaluated. Wild-type and *OsDIRP1*-overexpressing rice plants were grown for 5 weeks under normal conditions and then irrigated with water containing 200 mM NaCl. After 16–18 days of salt treatment, these plants were transferred back to normal irrigation conditions and were allowed to grow for at least 1 month to recover, and their growth patterns were monitored. As shown in Figures 4A,B, the *Ubi:OsDIRP1-sGFP* transgenic lines displayed more evident developmental anomalies with markedly retarded growth as compared to the wild-type plants, and the survival rates of the *Ubi:OsDIRP1-sGFP* lines after salt treatment were 27.6 ± 5.7% – 47.9 ± 7.8%, while that of the wild-type plants was 72.6 ± 8.9%.

A leaf senescence assay was performed with leaf disks (0.5 cm in diameter) prepared from 5-week-old rice plants. The leaf disks were floated in a solution supplemented with different concentrations (0, 200, 400, 600, and 800 mM) of NaCl for 3 days under long-day conditions (16 h light and 8 h dark). The results showed that the wild-type leaf segments retained approximately 78%, 50%, 38%, and 21% of their total leaf chlorophyll content when they were exposed to 200, 400, 600, and 800 mM NaCl, respectively (Figures 4C,D). On the other hand, the transgenic leaf segments were more sensitive to high salinity than the wild-type leaves, and approximately 55–74%, 19–22%, 14–16%, and 10–14% of the total leaf chlorophyll content was retained in response to 200, 400, 600, and 800 mM NaCl, respectively (Figures 4C,D). These results indicated that the *OsDIRP1-*overexpressing plants are less tolerant to high salinity than the wild-type plants.

We next examined the phenotype of *Ubi:RNAi-OsDIRP1* knock-down transgenic plants in response to dehydration. Unlike the overexpressing lines, the T4 *RNAi* (independent lines #1, #2, and #3) plants were very similar to the wild-type plants in terms of drought stress tolerance (Supplementary Figure S3). We repeated this experiment, but failed to detect a difference in tolerance to drought stress between the wild-type and *Ubi:RNAi-OsDIRP1* plants. These results led us to hypothesize that incomplete suppression of *OsDIRP1* may not have detectable effects on stress tolerance. Alternatively, other RING E3 ligase homologs may have complemented the phenotype of the *Ubi:RNAi-OsDIRP1* knock-down plants. Overall, our phenotypic analysis revealed that overexpression of *OsDIRP1* reduced tolerance to both drought (Figure 3) and high salinity (Figure 4) in rice plants. With this in mind, we concluded that rice OsDIRP1 is a negative factor in the response to drought and salt stress.

### Decreased ABA Sensitivity of the *OsDIRP1*-Overexpressing Transgenic Rice Plants

**FIGURE 5:**
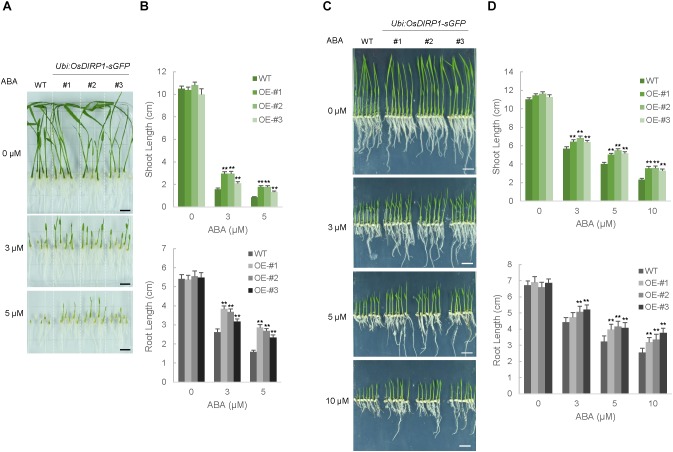
Hyposensitive phenotypes of *Ubi:OsDIRP1-sGFP* transgenic rice plants in response to ABA. **(A)** Germination tests of wild-type and T4 *Ubi:OsDIRP1-sGFP* (lines #1, #2, and #3) transgenic seeds in response to ABA. Sterilized seeds were germinated on half-strength MS medium in the presence of different concentrations (0, 3, and 5 μM) of ABA. Representative photos were taken at 7 days after germination. **(B)** Diagram of shoot and root lengths in the germination assays. The shoot and root lengths were measured at 7 days after germination. Data are means ± SE (*n* ≥ 3 biological independent experiments; >50 plants were used in each assay, ^∗∗^*P* < 0.01, Student’s *t*-test). **(C)** Post-germination assays. Wild-type and T4 *Ubi:OsDIRP1-sGFP* (lines #1, #2, and #3) seeds were germinated on half-strength MS medium for 2 days, after which germinated seedlings were transferred to half-strength MS medium supplemented with 0, 3, 5, and 10 μM ABA. Representative photos were taken at 6 days after transfer. **(D)** Diagram of the shoot and root lengths in the post-germination assays. Data are means ± SE (*n* ≥ 3 independent experiments; >100 plants were used in each assay, ^∗∗^*P* < 0.01, Student’s *t*-test).

To examine the role of OsDIRP1 in the response to ABA, ABA-dependent germination and post-germination assays were performed. Wild-type and T4 *Ubi:OsDIRP1-sGFP* (lines #1, #2, and #3) seeds were germinated on half-strength MS medium supplemented with 0, 3, and 5 μM ABA. After 7 days of incubation, the shoot and root lengths were measured. Without ABA, the germination rates of the wild-type and *OsDIRP1*-overexpressors were very similar (Figures 5A,B). In contrast, these genotypes were easily distinguishable in the presence of ABA. With 3 μM ABA, the shoot and root lengths of the wild-type seedlings were 1.6 ± 0.1 cm and 2.6 ± 0.2 cm, respectively, while those of *Ubi:OsDIRP1-sGFP* were 2.1 ± 0.1 cm – 3.0 ± 0.2 cm and 3.2 ± 0.1 cm – 3.8 ± 0.1 cm, respectively. These differences became even more evident at the higher concentration of ABA. In the presence of 5 μM ABA, the wild-type shoot and root lengths were 0.9 ± 0.1 cm and 1.6 ± 0.1 cm, respectively (Figures 5A,B). However, the shoot and root lengths of the *Ubi:OsDIRP1-sGFP* seedlings were 1.3 ± 0.1 cm – 1.8 ± 0.1 cm and 2.3 ± 0.1 cm – 2.9 ± 0.2 cm, respectively. These results indicated that the *OsDIRP1*-overexpressors were hyposensitive to ABA relative to the wild-type seedlings during the germination stage.

For the post-germination assay, wild-type and T4 *Ubi:OsDIRP1-sGFP* seeds were germinated on half-strength MS medium for 2 days and then transferred to medium containing 0, 3, 5, and 10 μM ABA. Then, the growth of these seedlings was monitored for 6 days after transfer. Consistent with the results of the germination assay, the *OsDIRP1*-overexpressing seedlings appeared to be ABA-insensitive during post-germination growth. For example, in the presence of 10 μM ABA, the shoot lengths of the wild-type and *Ubi:OsDIRP1-sGFP* plants were 2.3 ± 0.2 cm and 3.3 ± 0.2 cm – 3.6 ± 0.2 cm, respectively (Figures 5C,D). Further, the root lengths of these plants were 2.5 ± 0.3 cm and 3.2 ± 0.3 cm – 3.8 ± 0.3 cm, respectively, with 10 μM ABA. Thus, the *OsDIRP1*-overexpressors were insensitive to ABA during both the germination and post-germination stages, which is in agreement with the view that OsDIRP1 plays a negative role in the response to ABA. Taken together, the results of the phenotypic analyses in Figures 3–5 suggest that OsDIRP1 acts as a negative factor in the ABA-mediated drought and high salt responses in rice.

### *OsDIRP1*-Overexpressors Exhibited Enhanced Tolerance to Cold Stress Compared to Wild-Type Plants

**FIGURE 6:**
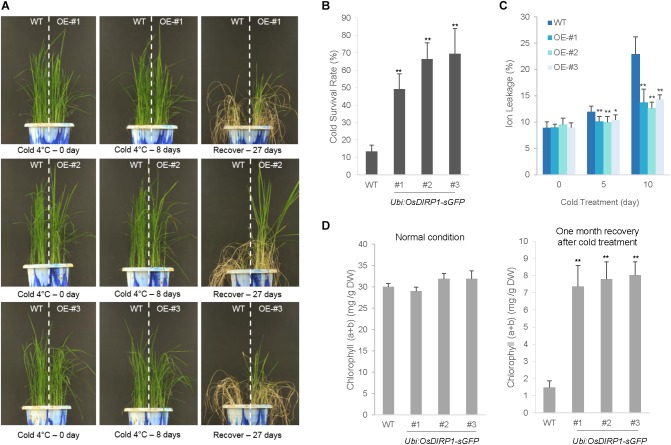
Increased tolerance of *Ubi:OsDIRP1-sGFP* transgenic rice plants to cold stress. **(A)** Cold stress phenotypes of wild-type and T4 *Ubi:OsDIRP1-sGFP* transgenic plants. Light-grown, 5-week-old wild-type and T4 *Ubi:OsDIRP1-sGFP* (lines #1, #2, and #3) plants were transferred to a cold room at 4°C for 8 days, after which the plants were recovered at 28°C for 27 days. OE represents *OsDIRP1-*overexpressing transgenic plants. **(B)** Survival rates of wild-type (WT) and T4 *Ubi:OsDIRP1-sGFP* plants in response to cold stress. Data are means ± SE (*n* ≥ 6 independent biological experiments; >30 plants were used in each assay, ^∗∗^*P* < 0.01, Student’s *t*-test). **(C)** Electrolyte leakage analysis of wild-type (WT) and T4 *Ubi:OsDIRP1-sGFP* plants in response to cold stress. Electrolyte leakage analysis was conducted using 8-day-old wild-type and transgenic seedlings at different time points before and after cold (4°C) treatment (0, 5, and 10 days). Data are means ± SD (*n* = 3 independent biological experiments; 12 plants of each genotype were used in each experiment, ^∗^*P* < 0.05, ^∗∗^*P* < 0.01, Student’s *t*-test). **(D)** Total leaf chlorophyll content of wild-type and T4 *Ubi:OsDIRP1-sGFP* (lines #1, #2, and #3) transgenic rice plants before and after cold treatment. The amounts of leaf chlorophyll (chlorophyll a + chlorophyll b) of mock-treated (before cold) and cold-treated plants were determined 1 month after recovery from cold stress. Data are means ± SE (*n* ≥ 3 biological independent experiments; 30 plants were used in each assay, ^∗∗^*P* < 0.01, Student’s *t*-test).

Because the *Ubi:OsDIRP1-sGFP* lines were hypersensitive to drought and high salinity stress, we considered the possibility that *Ubi:OsDIRP1-sGFP* lines may also exhibit altered tolerance to low temperature stress. To examine this possibility, wild-type and T4 *Ubi:OsDIRP1-sGFP* (lines #1, #2, and #3) plants were grown at 28°C for 5 weeks under long-day conditions (16 h light and 8 h dark) and subsequently subjected to cold stress by transferring them to a cold room at 4°C under continuous light. After 8 days of low temperature treatment, the plants were transferred back to the growth room at 28°C and their growth patterns were monitored. Under our experimental conditions, most of the wild-type plants exhibited anomalous growth patterns, with pale green and yellowish leaves, after recovery from cold stress. They were unable to grow, and most eventually died, with a survival rate of 13.4 ± 3.6% (Figures 6A,B). In contrast, the *OsDIRP1*-overexpressors were clearly healthier, with higher survival rates (49.3 ± 8.7% for line #1, 66.5 ± 9.3% for line #2, and 69.4 ± 14.6% for line #3) than the wild-type plants.

An electrolyte leakage analysis was conducted using 8-day-old seedlings before and after cold stress treatment. Wild-type and *OsDIRP1*-overexpressing seedlings were soaked in 35 mL of distilled water at 4°C, and the rates of electrolyte leakage were measured at different time points (0, 5, and 10 days). As shown in Figure 6C, the *Ubi:OsDIRP1-sGFP* seedlings exhibited lower ion leakage (10.0 ± 1.0% – 10.4 ± 1.0% at 5 days and 12.7 ± 1.1% – 14.3 ± 0.9% at 10 days) than the wild-type seedlings (12.0 ± 1.1% at 5 days and 22.9 ± 3.2% at 10 days) in response to prolonged cold stress. Consistently, the *OsDIRP1*-overexpressing progeny contained higher amounts of chlorophyll compared to that of the wild-type rice plants exposed to cold stress. After 1 month of recovery from cold treatment (4°C), the leaf chlorophyll content of the wild-type plants was 1.5 ± 0.4 mg/g DW, whereas that of the *Ubi:OsDIRP1-sGFP* plants was 7.3 ± 1.2 – 8.0 ± 0.8 mg/g DW (Figure 6D).

Finally, we examined the phenotypes of T4 *Ubi:RNAi-OsDIRP1* knock-down transgenic plants under low temperature stress. Similar to their drought phenotype, there was no evident phenotypic difference between the wild-type and *OsDIRP1 RNAi*-knock-down plants with regard to cold stress tolerance (Supplementary Figure S4).

### Cold-Responsive Genes Are Upregulated in *OsDIRP1*-Overexpressing Transgenic Rice Plants Compared to the Levels in Wild-Type Plants

Because *Ubi:OsDIRP1-sGFP* plants showed markedly enhanced tolerance to cold stress, we compared the expression patterns of cold-responsive genes in the wild-type and *Ubi:OsDIRP1-sGFP* plants by real-time qRT-PCR. The results showed that *OsDREB1A* and *OsDREB1B*, both of which are ABA-independent cold-responsive transcription factors (Dubouzet et al., 2003; Yamaguchi-Shinozaki and Shinozaki, 2006), were upregulated in *OsDIRP1*-overexpressing plants relative to the levels in wild-type plants after 24 h of cold treatment. In addition, the expression levels of ABA-independent cold stress-induced genes, such as *GAD* (*Os03g13300*; glutamate decarboxylase) and *MRP4* (*Os01g50100*; multidrug resistance protein 4) (Su et al., 2010), were also higher in *Ubi:OsDIRP1-sGFP* transgenic plants than in the wild-type plants under both normal and cold stress conditions. In contrast, the transcript level of *OsDREB1D* was slightly lower in the *OsDIRP1*-overexpressors than in the wild-type plants under low temperature condition. OsDREB1D is a CRT/DRE element-binding transcription factor that is involved in the ABA-dependent abiotic stress response (Zhang et al., 2009). Unlike other *DREB1* genes, *DREB1D* is induced by ABA, drought, and osmotic stress, but not by cold in *Arabidopsis* (Haake et al., 2002). These results suggested that elevation of ABA-independent cold-responsive gene expression in the *OsDIRP1*-overexpressors leads to increased tolerance to low temperature stress. Overall, these results indicated that, contrary to the drought and salt stress responses, *OsDIRP1*-overexpressing plants were more tolerant to prolonged cold stress than wild-type plants, suggesting that OsDIRP1 plays a positive role in the cold stress response in rice.

## Discussion

Considerable evidence has indicated that RING-type E3 Ub ligases modulate plant responses to a broad spectrum of abiotic stress (Lyzenga and Stone, 2012; Sadanandom et al., 2012). The majority of environmental stress-responsive RING E3 Ub ligases has been characterized in the dicot model plant *Arabidopsis*. In this study, a putative RING E3 ligase OsDIRP1 was identified and characterized in the monocot model cereal rice (Figure 1A). The low basal level of *OsDIRP1* expression was upregulated by drought, salt, and ABA treatments (Figures 1B,C). However, *OsDIRP1* expression was unchanged in response to low temperature, suggesting that *OsDIRP1* is differentially regulated by different abiotic stresses. Nuclear-localized RING E3s have been shown to up- or down-regulate the expression of stress-related genes (Dong et al., 2006; Qin et al., 2008; Catalá et al., 2011; Lim et al., 2013). Consistent with these results, OsDIRP1 was predominantly localized to the nucleus (Figure 1D), suggesting its role in regulating gene expression in response to abiotic stress.

Constitutive upregulation of *OsDIRP1* in transgenic rice plants (*Ubi:OsDIRP1-sGFP*) led to a severe decrease in tolerance to drought and high salinity, as evidenced by anomalous growth performance, reduced chlorophyll content, and more rapid leaf water loss rates, which resulted in markedly lower survival as compared to wild-type plants (Figures 2, 3). Furthermore, the *Ubi:OsDIRP1-sGFP* plants exhibited reduced sensitivities to exogenously supplied ABA during both the germination and post-germination stages (Figure 4). These observations suggest that OsDIRP1 participates in the negative feedback loop in the rice ABA-mediated drought and salt stress responses. In contrast, *Ubi:OsDIRP1-sGFP* showed increased tolerance to prolonged low temperature (4°C) treatment, as evidenced by stable growth, higher leaf chlorophyll content, and less electrolyte leakage relative to that in wild-type plants (Figure 5).

Given that plants are frequently exposed to a multitude of environmental stresses in different combinations at the same time, cross-talk between the cellular responses to individual stresses should occur in plant cells (Yamaguchi-Shinozaki and Shinozaki, 2006). The opposite roles of OsDIRP1 in the drought/salt and cold responses suggest that OsDIRP1 is involved in negative cross-talk between these stress tolerance mechanisms. Similar observation was reported for the *Arabidopsis* RING E3 ligase AtATL78. *RNAi-*mediated suppression of *AtATL78* conferred decreased drought tolerance and enhanced cold tolerance in *Arabidopsis* (Kim and Kim, 2013). In addition, transgenic rice plants that ectopically overexpressed *CaPUB1*, a hot pepper U-box E3 Ub ligase, were hypersensitive to drought but more tolerant to cold stress than wild-type rice plants (Min et al., 2016). Thus, it appears that the ubiquitination process conducted by a single E3 Ub ligase could confer opposite results in response to different stresses. This could be due to the fact that a single E3 ligase ubiquitinates different target proteins, depending on the cellular and physiological situations (Stone, 2018). However, the detailed mechanism underlying the opposite roles of OsDIRP1 remains to be determined.

**FIGURE 7:**
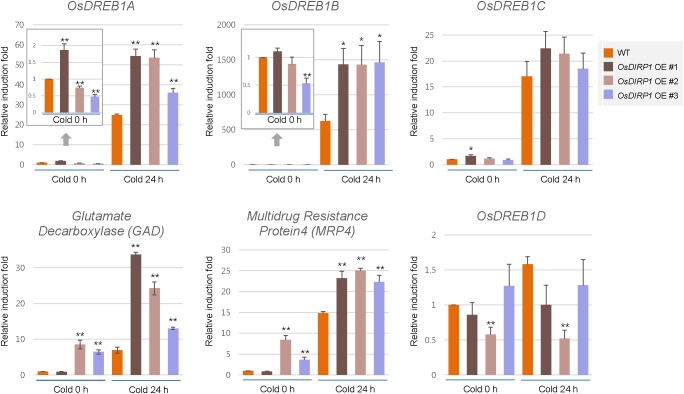
Expression analysis of cold stress-inducible genes in wild-type and *OsDIRP1*-overexpressing transgenic rice plants. Light-grown, 10-day-old wild-type and *Ubi:OsDIRP1-sGFP* transgenic plants were exposed to cold (4°C) stress for 0 or 24 h. The induction patterns of five stress-responsive genes, *OsDREB1A, OsDREB1B, OsDREB1D, GAD*, and *MRP4*, were analyzed by real-time qRT-PCR. The relative expression of each gene was normalized to that of *OsActin*. Data are means ± SE (^∗^*P* < 0.05, ^∗∗^*P* < 0.01, Student’s *t*-test) of three independent experiments.

The *OsDIRP1*-overexpressors displayed hypersensitivity to drought and salt stresses and hyposensitivity toward ABA (Figures 3–5), but they were tolerant to prolonged cold stress (Figure 6). Thus, we considered the possibility that the OsDIRP1-mediated cold response is ABA-independent. This hypothesis is supported, at least in part, by the finding that the ABA-independent cold-responsive genes (*OsDREB1A* and *OsDREB1B*), but not the ABA-dependent stress-related gene (*OsDREB1D*), were upregulated in *OsDIRP1*-overexpressing plants (Figure 7). Because OsDIRP1 is mainly localized to the nucleus, it would be a possible scenario that OsDIRP1 ubiquitinates a positive nuclear factor(s) in an ABA-dependent drought/salt response and a negative nuclear factor(s) in an ABA-independent cold response, which, in turn, suppresses drought/salt-responsive genes and activates cold-associated genes, respectively. To test this hypothesis, it is essential to identify the target proteins that are ubiquitinated by OsDIRP1. Thus, we are currently attempting to isolate the target proteins of OsDIRP1.

In addition to the *OsDIRP1*-overexpressing plants, *RNAi*-mediated knock-down transgenic rice plants (*Ubi:RNAi-OsDIRP1*) were also used for the phenotypic analysis. However, under our experimental conditions, these knock-down progeny did not show detectable phenotypic changes in response to water deficit and low temperature when compared to the wild-type plants (Supplementary Figures S3, S4). These results suggest that partial suppression of *OsDIRP1* failed to exert detectable effects on stress tolerance. In addition, it is likely that the phenotypes of *Ubi:RNAi-OsDIRP1* plants were rescued by other E3 Ub ligase(s). A database search revealed that Os06g47280 is the closest homolog of OsDIRP1. However, OsDIRP1 and Os06g47280 share relatively low amino acid identity (51%; Supplementary Figure S1). In addition, Os06g47280 lacks the conserved Cys and Arg residues in the C-terminus of the RING domain. In *Arabidopsis*, the RING E3 ligase AtAIRP2 plays a combinatory role with two other non-homologous RING E3 ligases, AtAIRP1 and AtSDIR1, and complements the drought- and salt-sensitive phenotypes of *atairp1* and *atsdir1* mutants, respectively (Qin et al., 2008; Cho et al., 2011; Oh et al., 2017). A similar situation may occur in these rice stress responses; thus, it is possible that an as-yet unknown E3 ligase complements the phenotypes of *Ubi:RNAi-OsDIRP1* knock-down plants.

Although OsDIRP1 possesses the C-terminal RING motif (Figure 1A), we were unable to detect *in vitro* E3 Ub ligase activity using a purified GST-OsDIRP1 protein (Supplementary Figure S5). This result raises two possibilities. First is that OsDIRP1 requires a post-translational modification, such as phosphorylation, for activation of its enzyme activity. In fact, OsDIRP1 is predicted to possess at least six putative phosphorylation sites (Supplementary Figure S6). Phosphorylation was shown to be required to activate *Arabidopsis* ICE1 (Inducer of CBF Expression), SINA2 (SEVER IN ABSENTIA), and RFP34/CHYR1 (RING ZINC-FINGER PROTEIN34/CHY ZINC-FINGER AND RING PROTEIN 1) E3 ligases as well as the rice OsPUB15 E3 Ub ligase (Ding Y. et al., 2015; Ding S. et al., 2015; Wang et al., 2015; Chen et al., 2018). Second, OsDIRP1 may not be a *bona fide* E3 ligase, but instead may play an unidentified role in response to environmental stimuli. It is worth noting that OsDIRP1 contains a putative beta-ketoacyl synthase active site in the N-terminal region (Figure 1). This second possibility, however, seems to be unlikely, as the RING motif in the OsDIRP1 is typical of many E3 ligases and highly homologous to that of other monocot RING E3 ligases (Supplementary Figure S1).

Rice plants frequently confront chilling injury at seedling or early vegetative stage when they are grown in early spring in temperate region, especially in the case of rainy day. In such growth conditions, it is a possible that rice plants need to turn on the mode of defense against cold stress and to repress the drought and salt responses. In conclusion, the data presented in this report indicate that OsDIRP1 is a negative regulator of drought and salt stress responses and a positive factor in the cold stress response in rice. These results further suggest that abiotic stress tolerance responses are subject to control by reciprocal and/or antagonistic cross-talk in the stress signaling pathways in higher plants.

## Author Contributions

LHC, HJM, MYB, and HGO performed the experiments. LHC, HJM, and WTK analyzed the data. LHC, HJM, and WTK designed the project and drafted the manuscript. WTK supervised the project and complemented the writing.

## Conflict of Interest Statement

The authors declare that the research was conducted in the absence of any commercial or financial relationships that could be construed as a potential conflict of interest.

## Footnotes

1 http://signal.salk.edu/cgi-bin/RiceGE
