# Supplementary material for: OsDIRP1, a Putative RING E3 Ligase, Plays an Opposite Role in Drought and Cold Stress Responses as a Negative and Positive Factor, Respectively, in Rice (Oryza sativa L.)
Source: Front Plant Sci. 2018 Dec 5;9:1797. doi: 10.3389/fpls.2018.01797 (PMC6290360; doi:10.3389/fpls.2018.01797)
Supplement: Supplementary file 1 [file Data_Sheet_1.PDF]

A

B

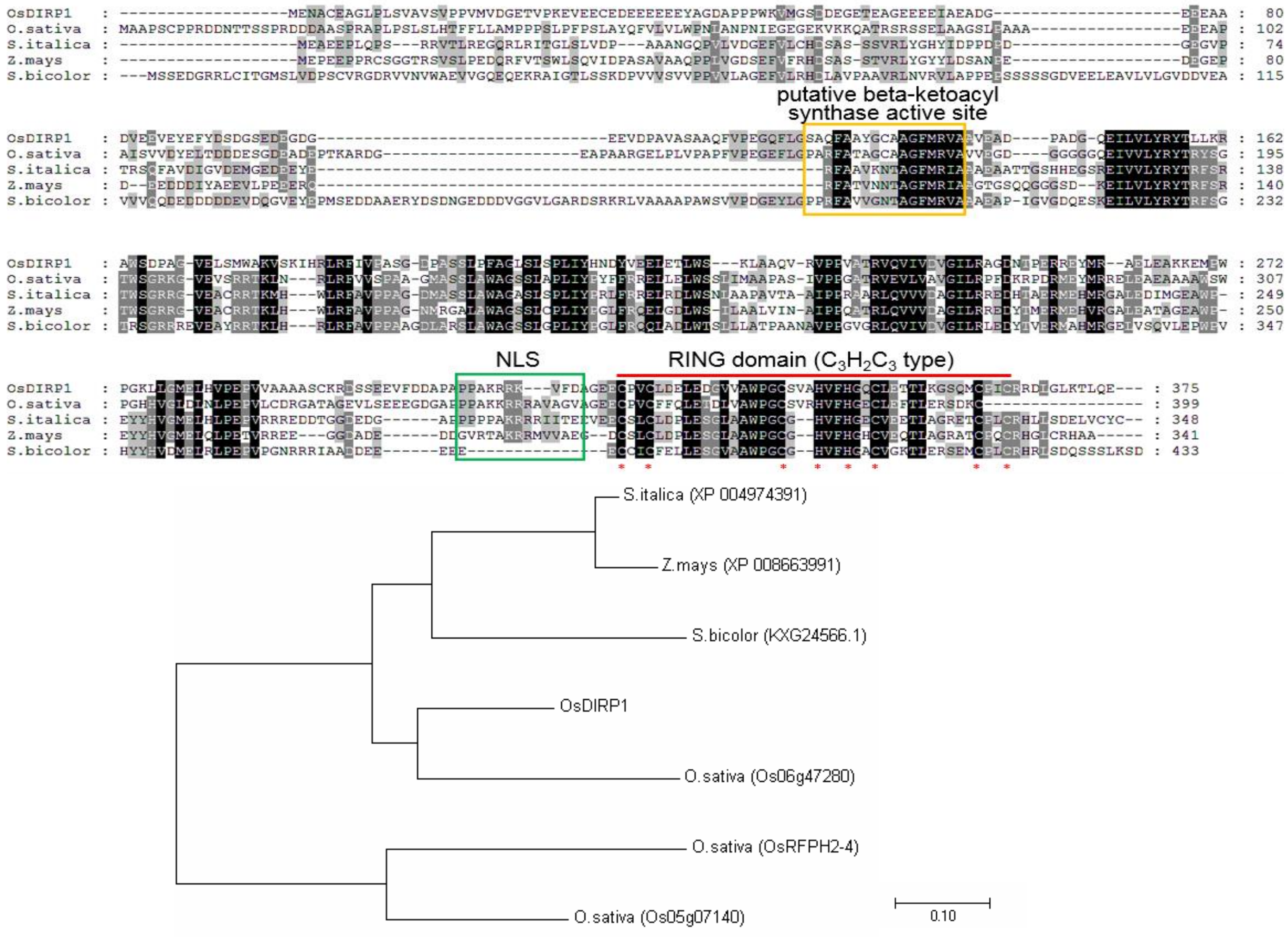

SUPPLEMENTARY FIGURE S1 | Sequence and phylogenetic analyses of OsDIRP1 homologs. (A) Comparison of the predicted amino acid sequences of OsDIRP1 homologs from different plant species. The derived amino acid sequence of OsDIRP1 was compared with those of other RING proteins from rice (*Oryza sativa* L.) (Os06g47280; GenBank accession No. BAD46054.1), millet (*Setaria italica*) (GenBank accession No. XP_004974391), maize (*Zea mays*) (GenBank accession No. XP_008663991), and sorghum (*Sorghum bicolor*) (GenBank accession No. KXG24566.1). The C-terminal $C_3H_2C_3$-type RING motif is indicated by a solid red line. The amino acid residues conserved in all five proteins are shown in black. Amino acid residues identical in at least three of the five sequences are shaded gray. The conserved Cys and His residues in the RING motif are indicated by asterisks. The nuclear localized sequence (NLS) and putative beta-ketoacyl synthase active site are boxed. **(B)** Phylogenetic analysis of OsDIRP1 homologs. Phylogenetic analysis was performed by the MEGA 7 program. OsRFPH2-4 is referred to as a RING finger protein, which has been known as an abiotic stress-related factor in rice.

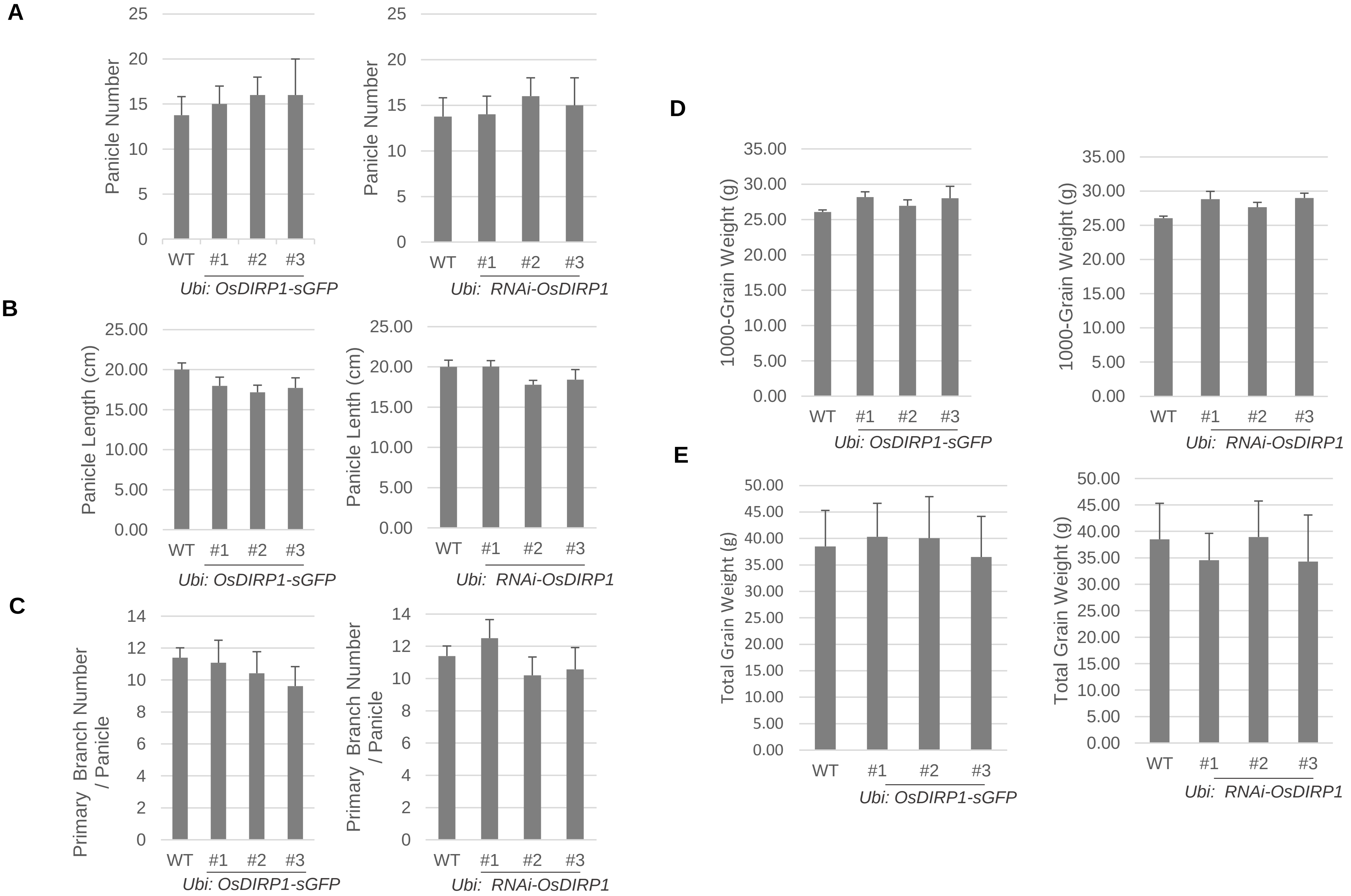

SUPPLEMENTARY FIGURE S2 | Agronomic traits of *Ubi:OsDIRP1-sGFP* and *Ubi:RNAi-OsDIRP1* T4 progeny grown in a paddy field. (A) Number of panicles per plant, **(B)** panicle length, **(C)** number of primary branches per panicle, **(D)** 1000-grain weight, and **(E)** total grain weight per plant. Data are means ±SD, estimated from 10 plants of each genotype.

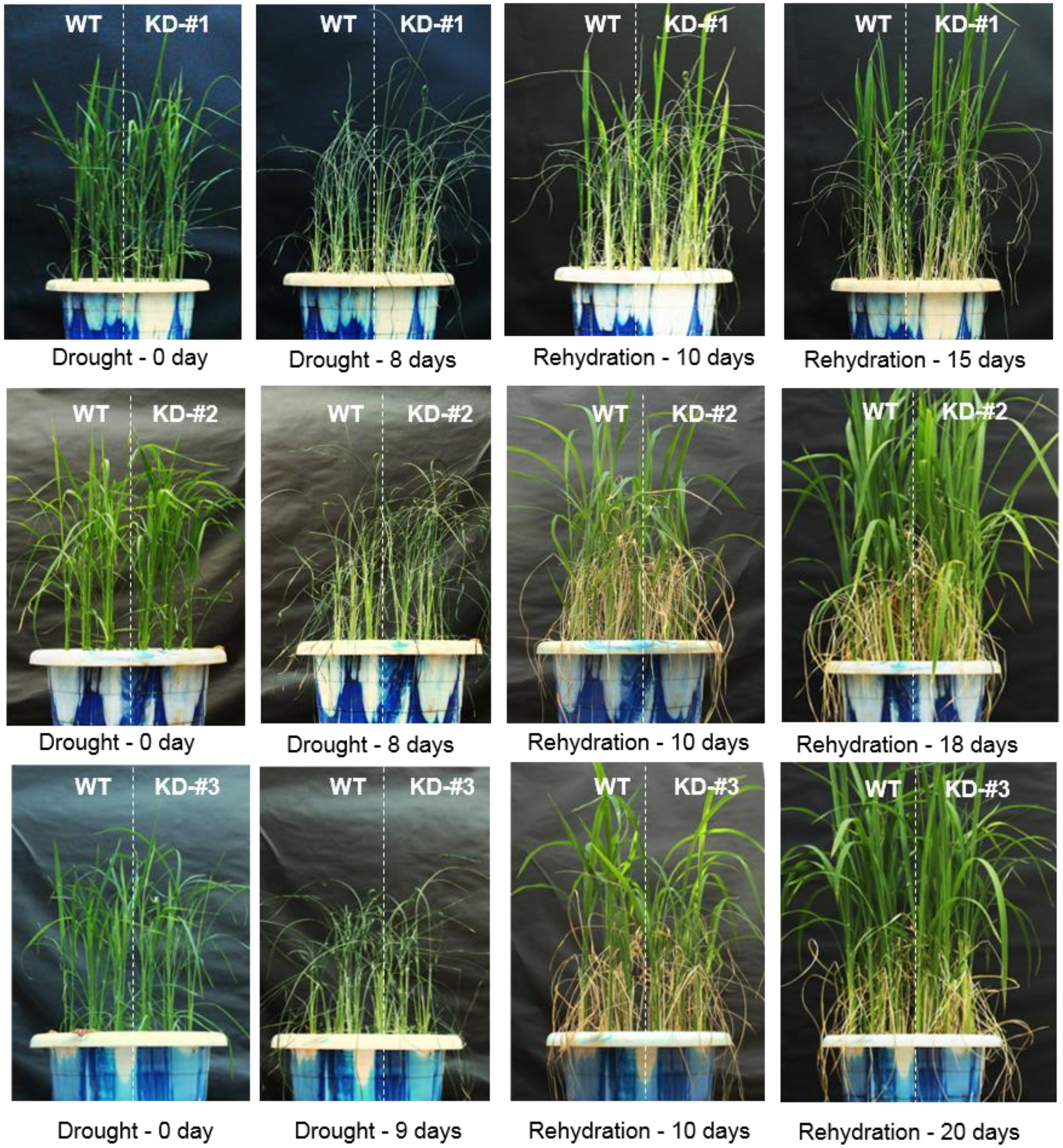

SUPPLEMENTARY FIGURE S3 | Phenotypic analysis of *Ubi:RNAi-OsDIRP1* knock-down transgenic plants in response to drought stress. Light-grown, 5-week-old wild-type (WT) and *Ubi:RNAi-OsDIRP1* (lines #1, #2, and #3) transgenic rice plants were grown without watering for 8-9 d (drought stress) and their survival was monitored for 15-20 d after rehydration. KD represents *RNAi*-mediated knock-down transgenic rice plants.

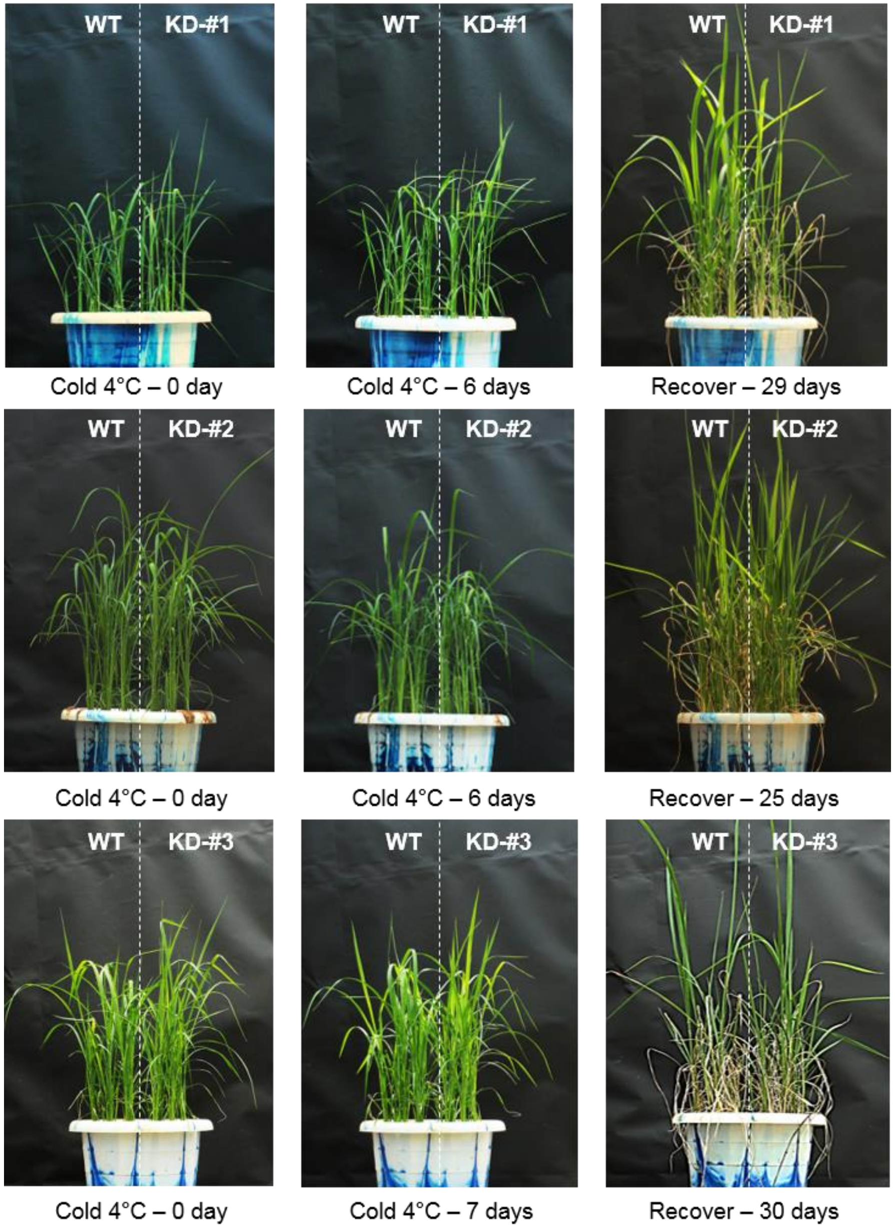

SUPPLEMENTARY FIGURE S4 | Phenotypic analysis of T4 *Ubi:RNAi-OsDIRP1* knock-down transgenic plants in response to cold stress. Light-grown, 5-week-old wild-type and *Ubi:RNAi-OsDIRP1* (lines #1, #2, and #3) plants were transferred to a cold room at 4°C for 6-7 d, after which the plants were recovered at 28°C, and their growth patterns were monitored. KD represents *RNAi*-mediated knock-down transgenic rice plants.

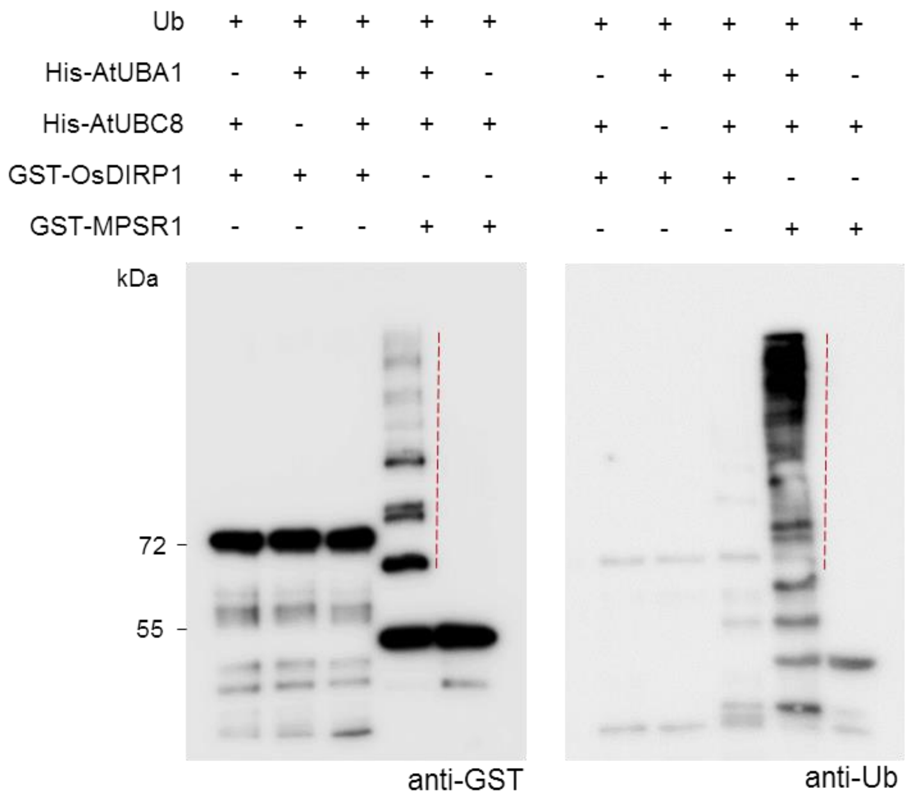

SUPPLEMENTAL FIGURE S5 | *In vitro* self-ubiquitination assay of OsDIRP1. Recombinant GST-OsDIRP1 protein was incubated in ubiquitination reaction buffer at 30°C for 1 h in the presence or absence of E1 (*Arabidopsis* UBA1) and E2 (*Arabidopsis* UBC8). The reaction mixtures were subjected to immunoblot analysis with anti-GST and anti-Ub antibodies. *Arabidopsis* RING E3 Ub ligase MPSR1 (Kim et al., 2017) was used as a positive control for E3 Ub ligase. Vertical dashed lines indicate high-molecular mass self-ubiquitinated ladders.

MENACEAGLPLSVAVSVPPVMVDGETVPKEVEECEDEEEEEE**Y**AGDAPPP
WKVMGSDDEGETEAGEEEEIAEADGEEEAADVEEVE**Y**EFYD**S**DG**S**EDEGD
GEEVDPAVASAAQFVPEGQFLGSAQFAAYGCAAGFMRVAAVEADPADGQE
ILVLYRYTLLKRAWSDPAGVELSMWAKVSKIHRLRFIVPASGDPASSLPF
AGLSLSPLIYHNDYVEELETLWSKLAAQVRVPPVATRVQVIVDVGILRAG
DNTPERREYMRAELEAKKEMPWPGKLLGMELHVPEPVVAAAASCKRD**SS**E
EVFDDAPAPPAKRRKVFDAGEECPVCLDELEDGVVAWPGCSVAHVFHGQC
LETTLKGSQMCPICRRDLGLKTLQE

SUPPLEMENTAL FIGURE S6 | Putative phosphorylation sites of OsDIRP1. Potential phosphorylation motifs in OsDIRP1, as predicted by NetPhos 3.1 software (http://www.cbs.dtu.dk/services/NetPhos/), are marked in red.
