# Supplementary material for: OsDIRP1, a Putative RING E3 Ligase, Plays an Opposite Role in Drought and Cold Stress Responses as a Negative and Positive Factor, Respectively, in Rice (Oryza sativa L.)
Source: Front Plant Sci. 2018 Dec 5;9:1797. doi: 10.3389/fpls.2018.01797 (PMC6290360; doi:10.3389/fpls.2018.01797)
Supplement: Supplementary file 2 [file Data_Sheet_2.PDF]

SUPPLEMETAL TABLE S1. Nucleotide sequences of the primers used in this study.

Oligonucleotide name	Sequence	Purpose
OsDIRP1 CDS F	ATGGAGAACGCCTGCGAGG	Construction for transgenic plants
OsDIRP1 CDS R	CTACTCCTGCAAGGTTTTCAGGC	Gene cloning
OsDIRP1 CDS no stop R	CTCCTGCAAGGTTTTCAGGC	Construction for transgenic plants
OsDIRP1 RNAi F	CACCTGAAAACCTTGCAGGAGTAGA	Construction for transgenic plants
OsDIRP1 RNAi R	CAATGGATTGCTTATACTTAGCATCG	Construction for transgenic plants
OsDIRP1 RT F	TTGTGCCGGAAGGGCAGTTT	RT-PCR
OsDIRP1 RT R	TGTCGTAGAACTCGTACTCGACCTCC	RT-PCR
OsUbiquitin RT F	ATGCAGATCTTTGTGAAGACATTG	RT-PCR
OsUbiquitin RT R	TTACTGACCACCACGGAGGC	RT-PCR
DREB1B RT F	AGCTCGCCGGCTCCGACA	RT-PCR
DREB1B RT R	GGGAGAAATCTGGCACATTCC	RT-PCR
OsRab16b RT F	ACAAGGGCAACAACCACCAG	RT-PCR
OsRab16b RT R	GCTTGCAATGGCATCACAAG	RT-PCR
Hph probe F	ATGAAAAAGCCTGAACTCACC	Southern blot probe
Hph probe R	CTATTCCTTTGCCCTCGG	Southern blot probe
OsActin qRT F	CCCAAGGCTAACAGGGAGAA	qRT-PCR
OsActin qRT R	GACACCATCACCAGAGTCCA	qRT-PCR
OsDREB1A qRT F	CGACGACGACGAGGAGTC	qRT-PCR
OsDREB1A qRT R	TCACTCAGGACGTCCAGTTC	qRT-PCR
OsDREB1B qRT F	GGTCGTACTACGCGAGCTT	qRT-PCR
OsDREB1B qRT R	TAGTAGCTCCAGAGCGGCAT	qRT-PCR
OsDREB1C qRT F	CGTACTACGGCAACATGGAC	qRT-PCR
OsDREB1C qRT R	ATCTGCCAGCTCTGGTACTC	qRT-PCR
OsDREB1D qRT F	CGTTTCTGGATAGGCACGTC	qRT-PCR
OsDREB1D qRT R	CAGAGTCGGCGAAGTTGAG	qRT-PCR
MRP4 qRT F	CAGGCAGAGGAACAGGTGAT	qRT-PCR
MRP4 qRT R	CGTACCGGAACAAGCTGAAC	qRT-PCR
GAD qRT F	CGAGGAGCTCATCTTCCACA	qRT-PCR
GAD qRT R	TGGTAGTACTGGGCGATGAC	qRT-PCR
