# Supplementary material for: OsDIRP1, a Putative RING E3 Ligase, Plays an Opposite Role in Drought and Cold Stress Responses as a Negative and Positive Factor, Respectively, in Rice (Oryza sativa L.)
Source: Front Plant Sci. 2018 Dec 5;9:1797. doi: 10.3389/fpls.2018.01797 (PMC6290360; doi:10.3389/fpls.2018.01797)
Supplement: Supplementary file 3 [file Presentation_1.PPTX]

## Slide 1
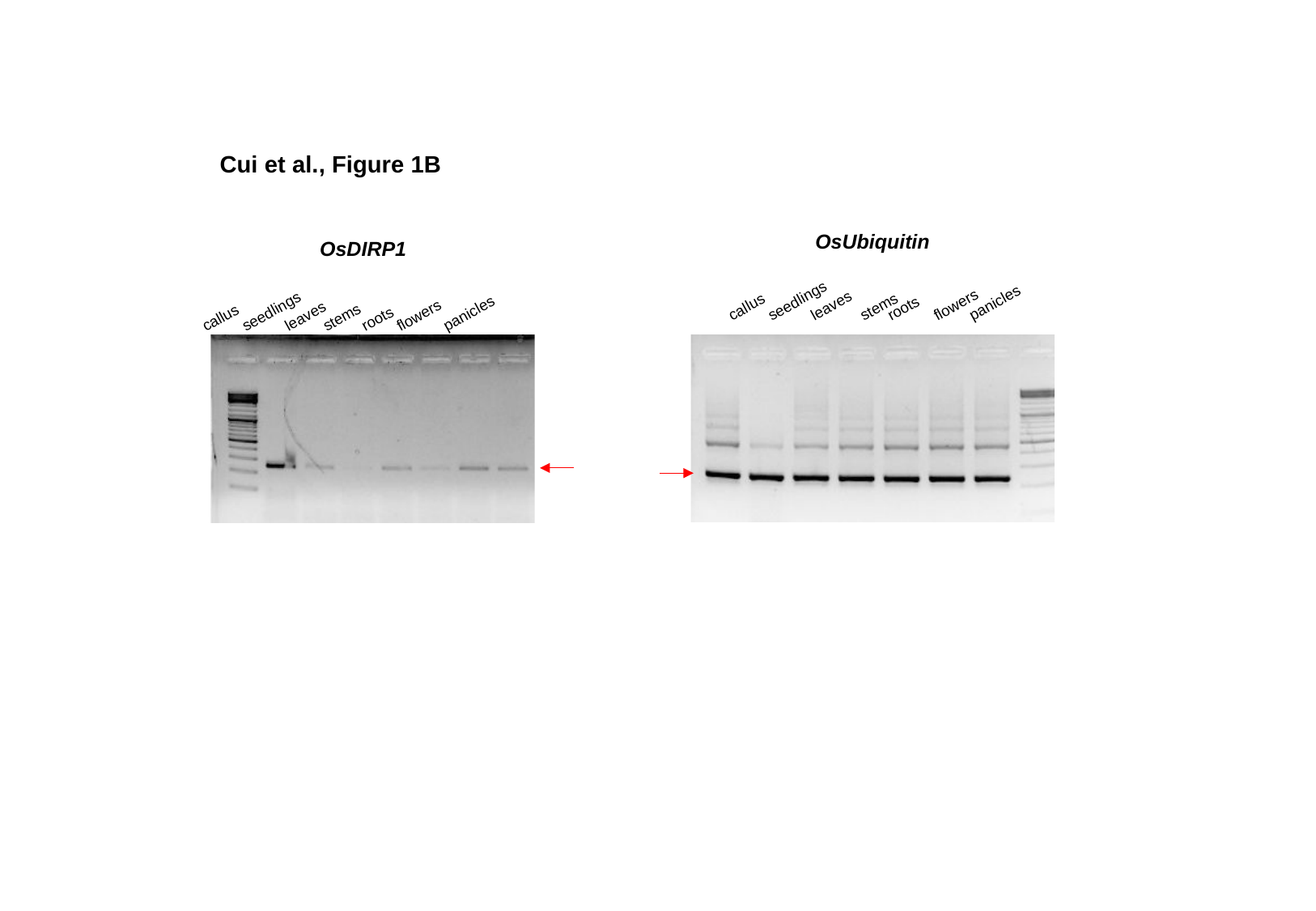

Cui et al., Figure 1B
OsUbiquitin
stems
callus
seedlings
flowers
roots
panicles
leaves
OsDIRP1
stems
callus
seedlings
flowers
roots
panicles
leaves

## Slide 2
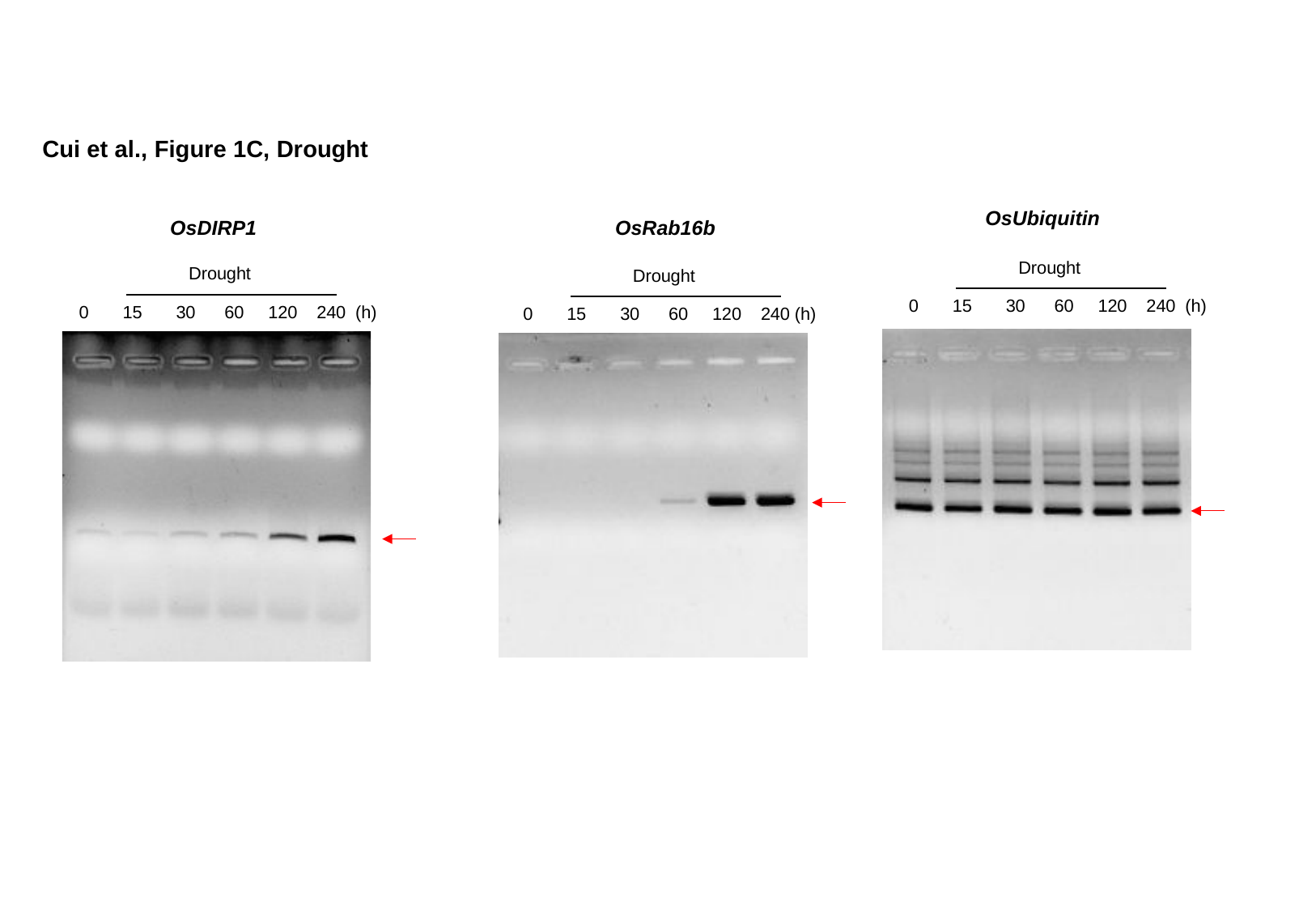

Cui et al., Figure 1C, Drought
OsUbiquitin
Drought
 0 15 30 60 120 240 (h)
OsDIRP1
Drought
 0 15 30 60 120 240 (h)
OsRab16b
Drought
 0 15 30 60 120 240 (h)

## Slide 3
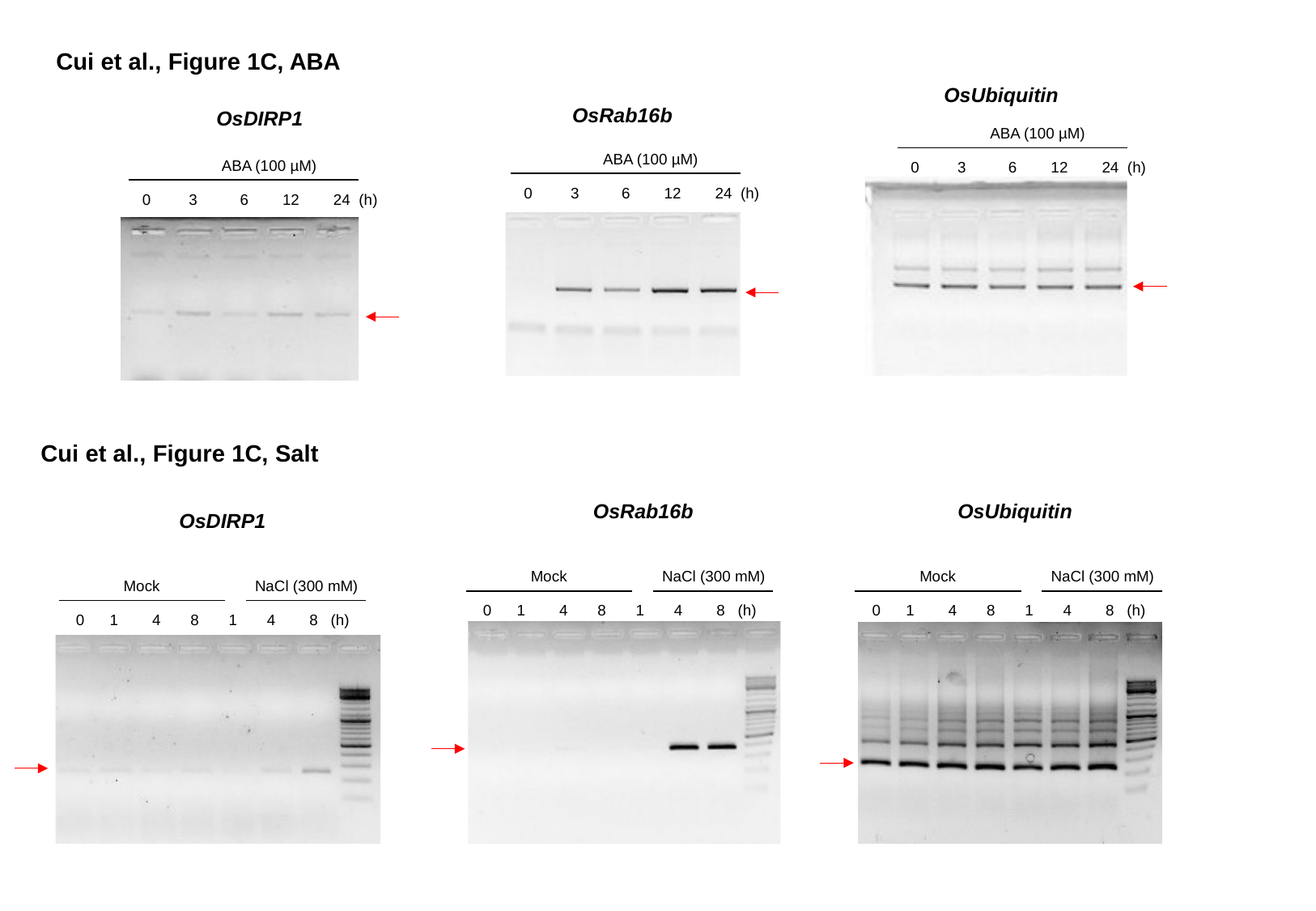

Cui et al., Figure 1C, ABA
OsUbiquitin
ABA (100 µM)
 0 3 6 12 24 (h)
OsRab16b
ABA (100 µM)
 0 3 6 12 24 (h)
OsDIRP1
ABA (100 µM)
 0 3 6 12 24 (h)
Cui et al., Figure 1C, Salt
OsUbiquitin
Mock
NaCl (300 mM)
 0 1 4 8 1 4 8 (h)
OsRab16b
Mock
NaCl (300 mM)
 0 1 4 8 1 4 8 (h)
OsDIRP1
Mock
NaCl (300 mM)
 0 1 4 8 1 4 8 (h)

## Slide 4
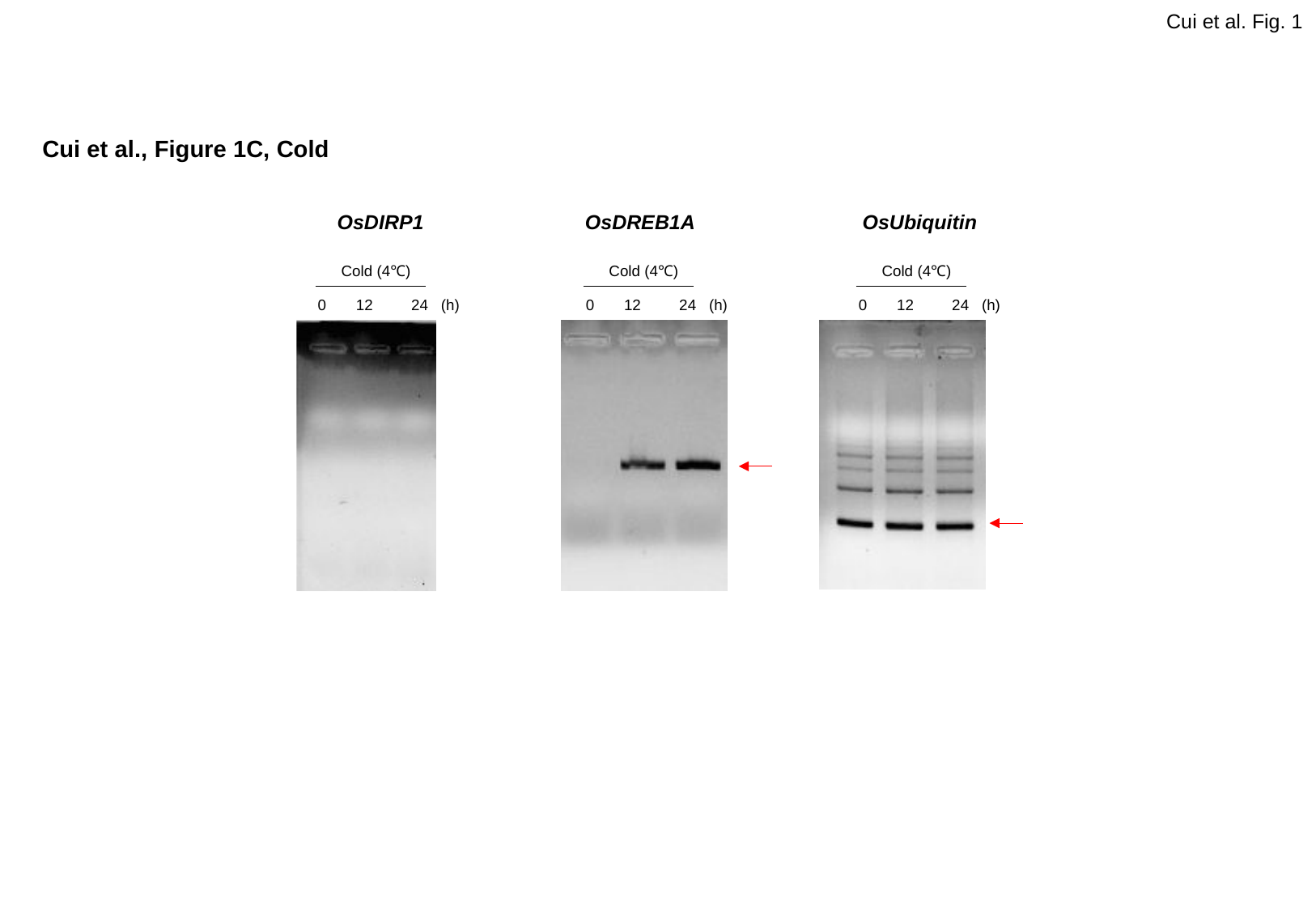

Cui et al. Fig. 1
Cui et al., Figure 1C, Cold
OsDIRP1
OsDREB1A
OsUbiquitin
Cold (4℃)
Cold (4℃)
Cold (4℃)
0 12 24 (h)
0 12 24 (h)
0 12 24 (h)

## Slide 5
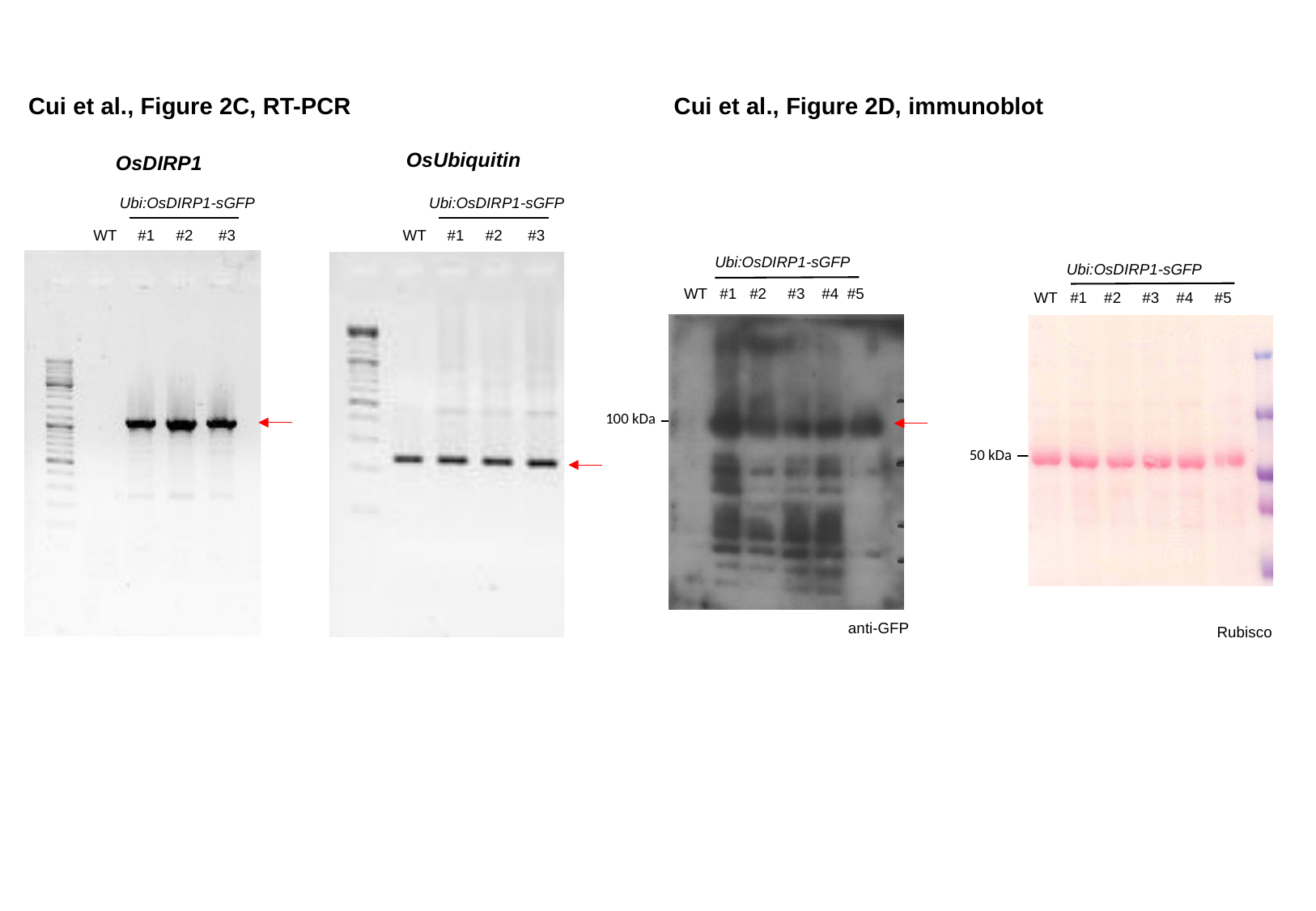

Cui et al., Figure 2D, immunoblot
Cui et al., Figure 2C, RT-PCR
OsUbiquitin
Ubi:OsDIRP1-sGFP
WT #1 #2 #3
OsDIRP1
Ubi:OsDIRP1-sGFP
WT #1 #2 #3
Ubi:OsDIRP1-sGFP
WT #1 #2 #3 #4 #5
100 kDa
anti-GFP
Ubi:OsDIRP1-sGFP
WT #1 #2 #3 #4 #5
50 kDa
Rubisco

## Slide 6
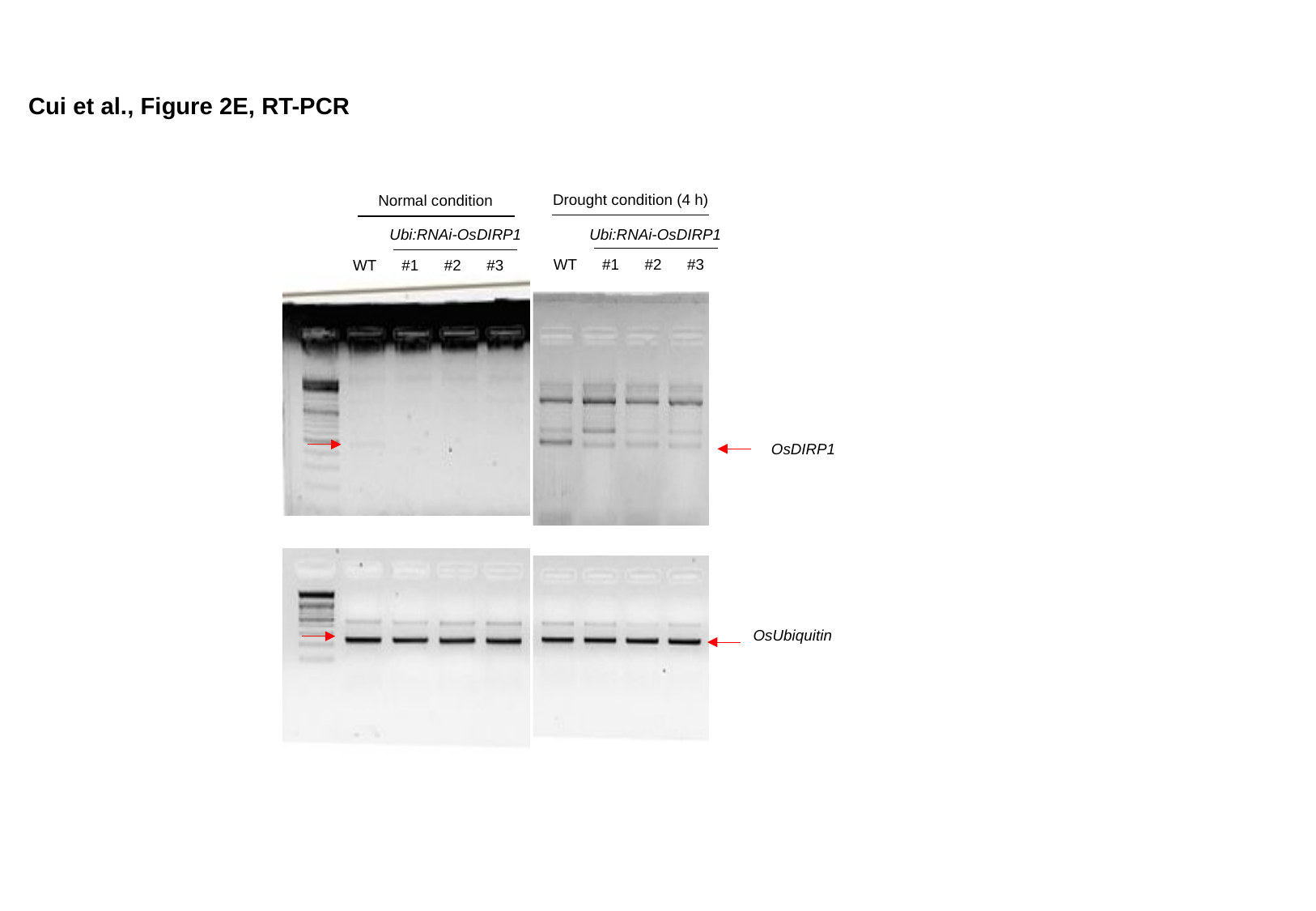

Cui et al., Figure 2E, RT-PCR
Drought condition (4 h)
Normal condition
Ubi:RNAi-OsDIRP1
Ubi:RNAi-OsDIRP1
WT #1 #2 #3
WT #1 #2 #3
OsDIRP1
OsUbiquitin
